# Nanotechnology based solutions for anti-leishmanial impediments: a detailed insight

**DOI:** 10.1186/s12951-021-00853-0

**Published:** 2021-04-15

**Authors:** Humzah Jamshaid, Fakhar ud Din, Gul Majid Khan

**Affiliations:** 1 Nanomedicine Research Group, Department of Pharmacy, Quaid-I-Azam University, Islamabad, 45320 Pakistan; 2 Islamia College University, Peshawar, Khyber Pakhtunkhwa Pakistan

**Keywords:** Leishmaniasis, Nanotechnology, Nano-DDS, Nano-adjuvants, Drug delivery, Mannosylated thiolated nanosystem, Transferosomes, Nanovaccines, Phyto-nano-DDS

## Abstract

As a neglected tropical disease, Leishmaniasis is significantly instigating morbidity and mortality across the globe. Its clinical spectrum varies from ulcerative cutaneous lesions to systemic immersion causing hyperthermic hepato-splenomegaly. Curbing leishmanial parasite is toughly attributable to the myriad obstacles in existing chemotherapy and immunization. Since the 1990s, extensive research has been conducted for ameliorating disease prognosis, by resolving certain obstacles of conventional therapeutics viz. poor efficacy, systemic toxicity, inadequate drug accumulation inside the macrophage, scarce antigenic presentation to body’s immune cells, protracted length and cost of the treatment. Mentioned hurdles can be restricted by designing nano-drug delivery system (nano-DDS) of extant anti-leishmanials, phyto-nano-DDS, surface modified—mannosylated and thiolated nano-DDS. Likewise, antigen delivery with co-transportation of suitable adjuvants would be achievable through nano-vaccines. In the past decade, researchers have engineered nano-DDS to improve the safety profile of existing drugs by restricting their release parameters. Polymerically-derived nano-DDS were found as a suitable option for oral delivery as well as SLNs due to pharmacokinetic re-modeling of drugs. Mannosylated nano-DDS have upgraded macrophage internalizing of nanosystem and the entrapped drug, provided with minimal toxicity. Cutaneous Leishmaniasis (CL) was tackling by the utilization of nano-DDS designed for topical delivery including niosomes, liposomes, and transfersomes. Transfersomes, however, appears to be superior for this purpose. The nanotechnology-based solution to prevent parasitic resistance is the use of Thiolated drug-loaded and multiple drugs loaded nano-DDS. These surfaces amended nano-DDS possess augmented IC_50_ values in comparison to conventional drugs and un-modified nano-DDS. Phyto-nano-DDS, another obscure horizon, have also been evaluated for their anti-leishmanial response, however, more intense assessment is a prerequisite. Impoverished Cytotoxic T-cells response followed by Leishmanial antigen proteins delivery have also been vanquished using nano-adjuvants. The eminence of nano-DDS for curtailment of anti-leishmanial chemotherapy and immunization associated challenges are extensively summed up in this review. This expedited approach is ameliorating the Leishmaniasis management successfully. Alongside, total to partial eradication of this disease can be sought along with associated co-morbidities.

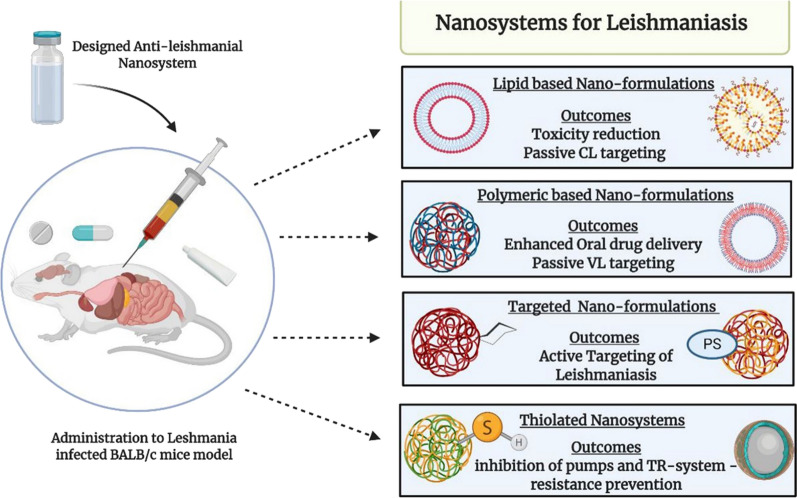

## Leishmaniasis—a grievous medical condition

Leishmaniasis, a neglected tropical disease, is the 3rd most challenging vector-borne disease. A huge number of cases of Leishmaniasis are reported annually all over the world. Additionally, according to the World Health Organization (WHO) published data, it has been reported that Leishmaniasis is endemic to approximately 100 countries of the world except for Polar Regions and Australia. Indeed, this disease is more pronounced in a tropical, subtropical, and temperate region and classified under the umbrella of neglected tropical disease as well [1–3]. According to 2012 stats, the death rate associated with visceral leishmaniasis (VL) was 10%-15% of the reported cases, worldwide [4]. Leishmaniasis is more prevalent in the poor population [5]. Most probably because of lower financial returns the pharmaceutical industries showed minimal concerns towards anti-leishmanials research and drug development. Worldwide, about 0.2–0.4 million cases of VL have been reported annually. However, 95% of the new VL cases usually outbreak in Sudan, South America particularly in Brazil, Kenya, Nepal, and in Sub-continent—India, and Bangladesh. Worldwide VL burden has been reduced significantly from 2012, which was 50,000–90,000 according to a 2017 data survey. Contrary to this, the annual registered cases of CL are around 0.7–1.2 million, across the globe. This form of Leishmaniasis usually appears mainly (85%) in Asian countries including Iraq, Iran, and Pakistan, in the South American region—in Brazil and Peru specifically, in Algeria, South Africa, Sudan, and Costa Rica [3, 6–8]. As far as Post Kalazar dermal Leishmaniasis is concerned, 50% of its new cases are reported in the East African region and 10% in the Indian Sub-continent. In American regions, genus *Lutzomyia* causes the spreading of the parasite, while the genus *Phlebotomus* acts as a vector in the rest of the world [7]. Leishmaniasis also hit Middle-East regions evidenced by a survey, reporting 1033 new CL cases in Lebanon in 2013, as compared to only 6 registered CL cases between 2000–2012 [9]. In India, Bihar is the hub of Leishmaniasis most probably due to pathetic poverty conditions (income below 1.0 USD/day). Since 2005, Nepal and India undergoing a campaign and disease control programs to minimize leishmanial burden by 1 infective case per 10,000 individuals at the sub-district level [5, 10]. A review published in 2006 stated that Leishmaniasis holds a high treatment cost of 30–1500 USD, indeed, a challenging factor for poverty. Furthermore, the indirect treatment cost for Leishmaniasis in Nepal was 53% of the total treatment cost. The total median treatment cost per patient in Srilanka was found to be 66.85 USD while the economic loss median cost of households was found to be 61.27 USD. The VL diagnostic and management cost in Nepal is more than the median annual domestic income [11].

About 20 different species of trypanosomatid protozoans belonging to the genus *Leishmania*, have been reported to cause Leishmaniasis [1]. Moreover, the old and new world Leishmaniasis is characterized primarily by the causative species. Various Leishmania species such as *L. tropica*, *L. infantum*, and *L. major* are the primary causative means for Old World Leishmania. Whereas, *L. mexicana, L. braziliensis*, and *L. viannia* are mainly responsible for New World Leishmania [12]. VL is mainly caused by *L. donovani, L. infantum* and *L. tropica*. However, the major causative species for CL are *L. tropica*, *L. major, L. infantum, L. braziliensis, L. amazonensis,* and *L. guyanensis* [1, 2, 12, 13]. The principal vector responsible for the mammal to human transmission of the leishmanial parasite is the Phlebotomine sandfly. On taxonomic grounds, it has been concluded by Lane that among 6 genera of the Phlebotomine sub-family, only 2 genera are chiefly associated with leishmanial dissemination. Furthermore, *Phlebotomus* genera are the chief vector for old world Leishmania and *Lutzomyia* genera are associated with New world Leishmania spreading [1, 2]. Another possible reason for the prevalence of Leishmaniasis in the Mediterranean/tropical region is the hyperactivity of sand fly in warmer and humid areas [14].

**Fig. 1:**
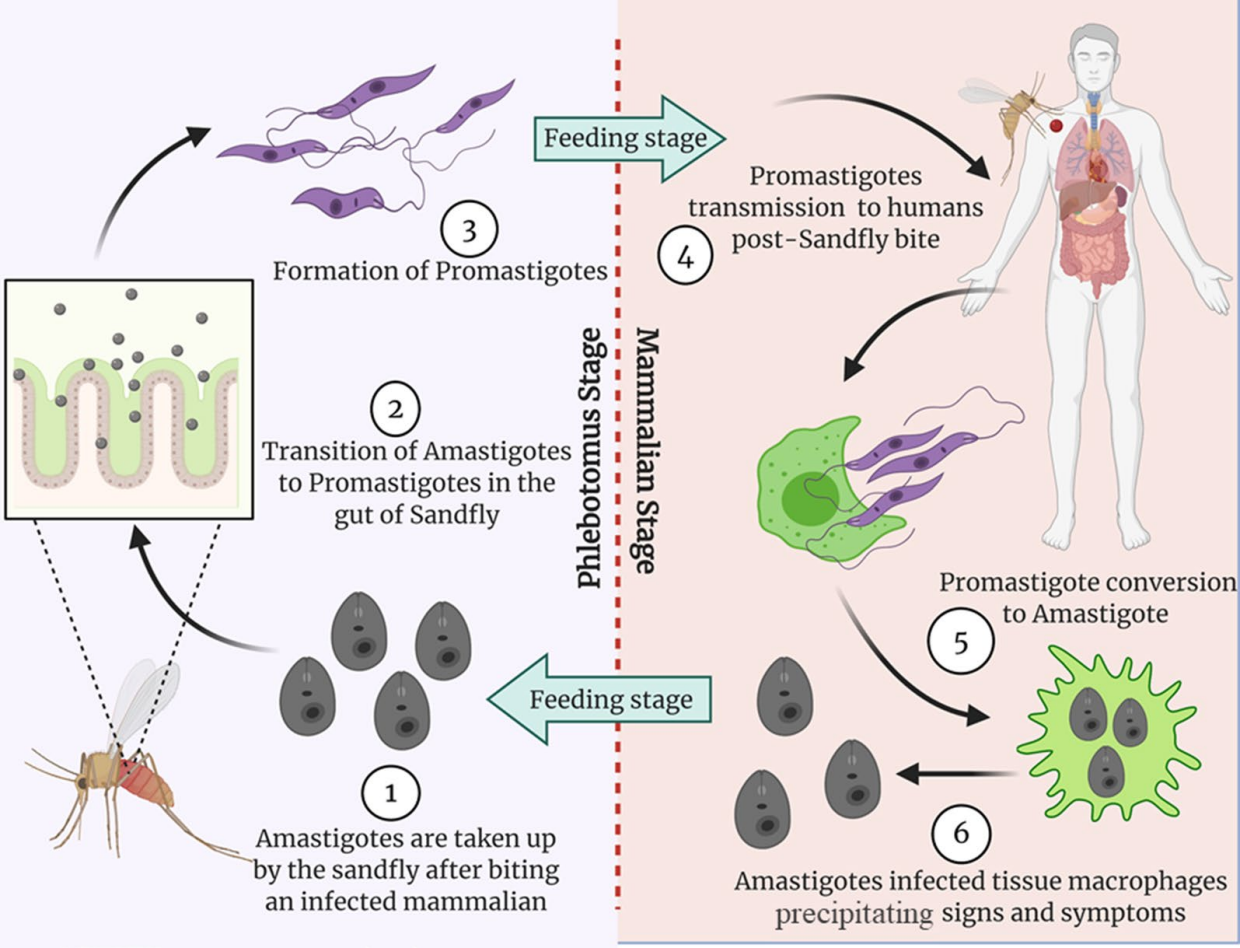
Life Cycle of Leishmania and its stages; [1] Sandfly acquire amastigotes from infected mammalian host; [2] Amastigotes conversion into the promastigotes in sandfly gut; [3, 4] Transmission of promastigotes from sandfly to the healthy mammalian host by sandfly bite; [5, 6] Promastigotes conversion to amastigotes which infect the macrophages and induce a symptomatic condition

Principle reservoirs for VL induced by *L. donovani* strain are the humans and its transmission is usually occurring from 700 m below sea level to the altitude of 1000 m, particularly in the Sub-continent, Nepal, and Bhutan [15–17]. This form usually outbreaks in villages with high poverty rates and mud-walled houses. Moreover, the presence of cattle under the same roof or nearby places favored the spreading of parasites via *Phlebotomus argentipes* [2]. The primary reservoir of leishmanial protozoa (*L. infantum*) is the wild mammals like hare, dogs, and the successful infection development requires the appropriate anthroponotic or zoonotic transmission of the parasite to the human host. Non-vector parasitic transmitting ways are also described which includes organ transplantation, blood products transfusion, and laboratory mishaps. However, the CL spreading by these routes is rare. Male gender, meager living facilities, and younger age below 15 are more prone to CL infection [2]. Two distinct morphological forms of Leishmania have been identified in its life cycle, which are the promastigote and amastigote. The former form is long, flagellated, and usually present extracellular in the vector, sandflies. However, the latter one is small in size, aflagellated and present inside the monocytes/macrophages of the human or mammalian host [1]. A detailed leishmanial life cycle is illustrated in Fig. 1.

**Fig. 2:**
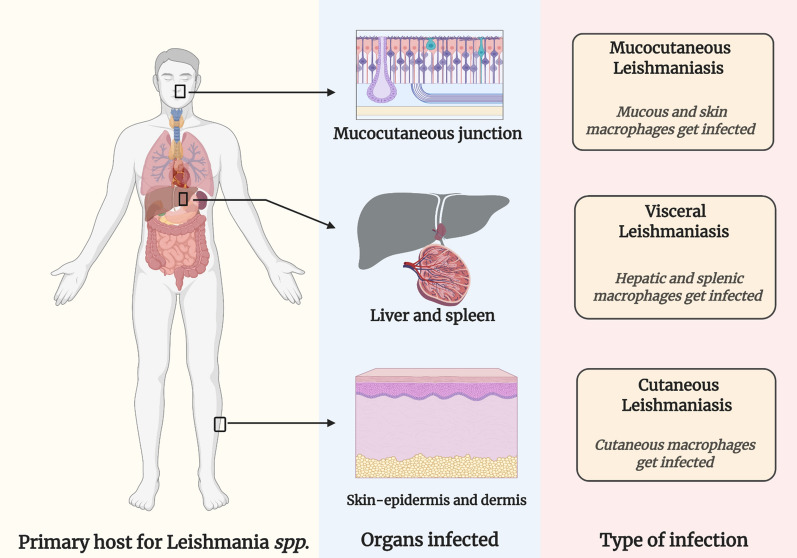
Types of leishmaniasis and the organs affected; Mucocutaneous Leishmaniasis—mucocutaneous junctions get infected, Visceral Leishmaniasis—Liver and spleen get infected, Cutaneous Leishmaniasis—skin gets infected

**Fig. 3:**
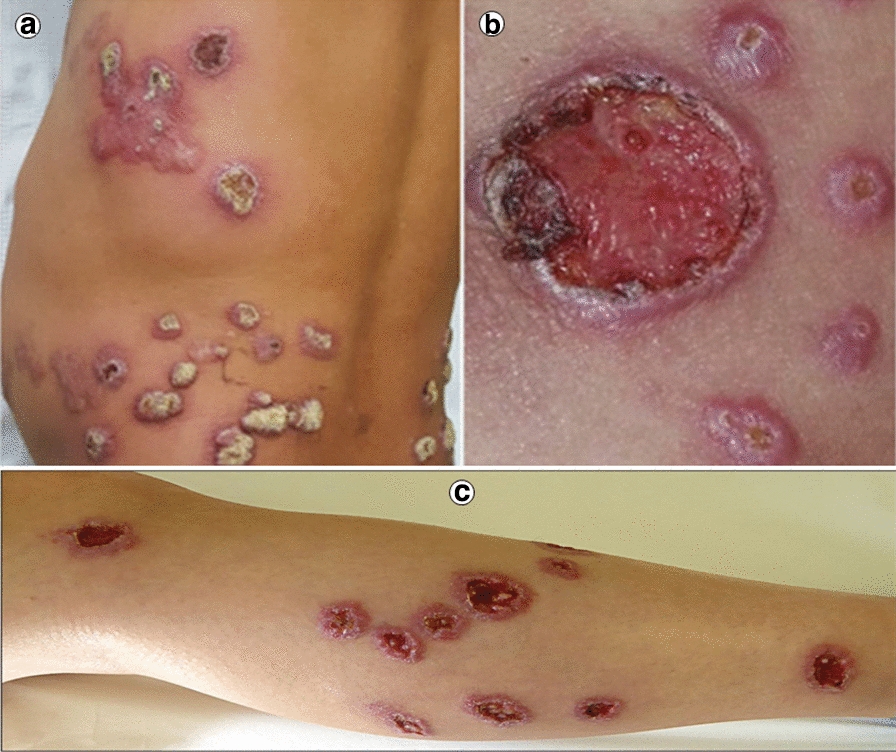
**a** Keratitis with diffused nodular lesion [24]. **b** Ulcerated lesion with crusty border with non-bleeding erythematous center [24]. **c** Multiple CL lesions on the right leg of 27-years old women [25]

Depending upon the severity of the affected macrophages, the infection ranges from asymptomatic to clinically evident disorder. The symptomatic infection is either localized to the skin or mucocutaneous junction, termed as CL and mucocutaneous Leishmaniasis (MCL), respectively. Whereas, systemic infection is referred to as VL [18]. Thus, because of clinical signs and symptoms, Leishmaniasis is usually presented in three forms as shown in Fig. 2. VL usually manifests with irregular hyperthermic episodes accompanied with spleno-hepatomegaly and blood dyscrasia-pancytopenia and hyper gammaglobulinemia appear. Weight loss is also common in VL patients, in particular, children [9, 19]. Wasting and acute malnutrition encounter, but it is uncertain whether a consequence of elevated parasitic burden or poverty [19]. A surge in adrenocorticotropic hormone and cortisol due to excessive cytokine release imparts skin hyperpigmentation in VL individuals, which in the Indian native language, is termed as “Kalazar-a black fever” [20]. The incubation duration of the leishmanial parasite ranges from 7 to 147 days [21]. Hence, the commencement of VL is either acute or insidious. VL infection, unfortunately, can be lethal on account of serious anemic conditions or secondary bacterial infection and sepsis [22]. Contrary to VL, CL is a non-lethal skin infection that reduces a patient’s life quality, impart psychological problems and social stigmatism [23]. The lesion morphology varies with the infective species. In general, at the initial stages of infection, the papule develops which gradually transforms into an ulcerative nodule over an extended duration of few months. Lesion site is not limited to face and extremities, because in diffused CL lesion develops at various cutaneous sites, illustrated in Fig. 3 [24, 25]. Lymphatic dissemination of the parasite, however, is probable. Lesions of *L. major* and *L tropica* infection are usually self-resolving, but unfortunately, instigate permanent cutaneous scarring. CL caused by *L. aethiopica* is quite dangerous owing to its diffused nature, deterioration, and dissemination of lesions to mucocutaneous junctions [26]. However, MCL manifestation is life-threatening and is believed to be encountered more frequently in the immunocompromised population [27, 28]. The major ulcer site in this form is an oronasal junction. As previously stated, MCL lesion can cause mutilation of nasal cartilage and nasal septum, which could ultimately cause nasal collapse [1, 29]. To summarize the discussion, it is conclusive that Leishmaniasis is a challenging medical condition and public health burden, globally. At the start of the last decade, Leishmaniasis was endemic to 98 countries but now the number is raised to 100. These facts are critical and demands global scientific attention towards mitigation of hurdles to attain its eradication.

## Challenges related to conventional anti-leishmanials—in the way of disease eradication

### Resistance emergence—a major setback for Leishmaniasis management

**Fig. 4:**
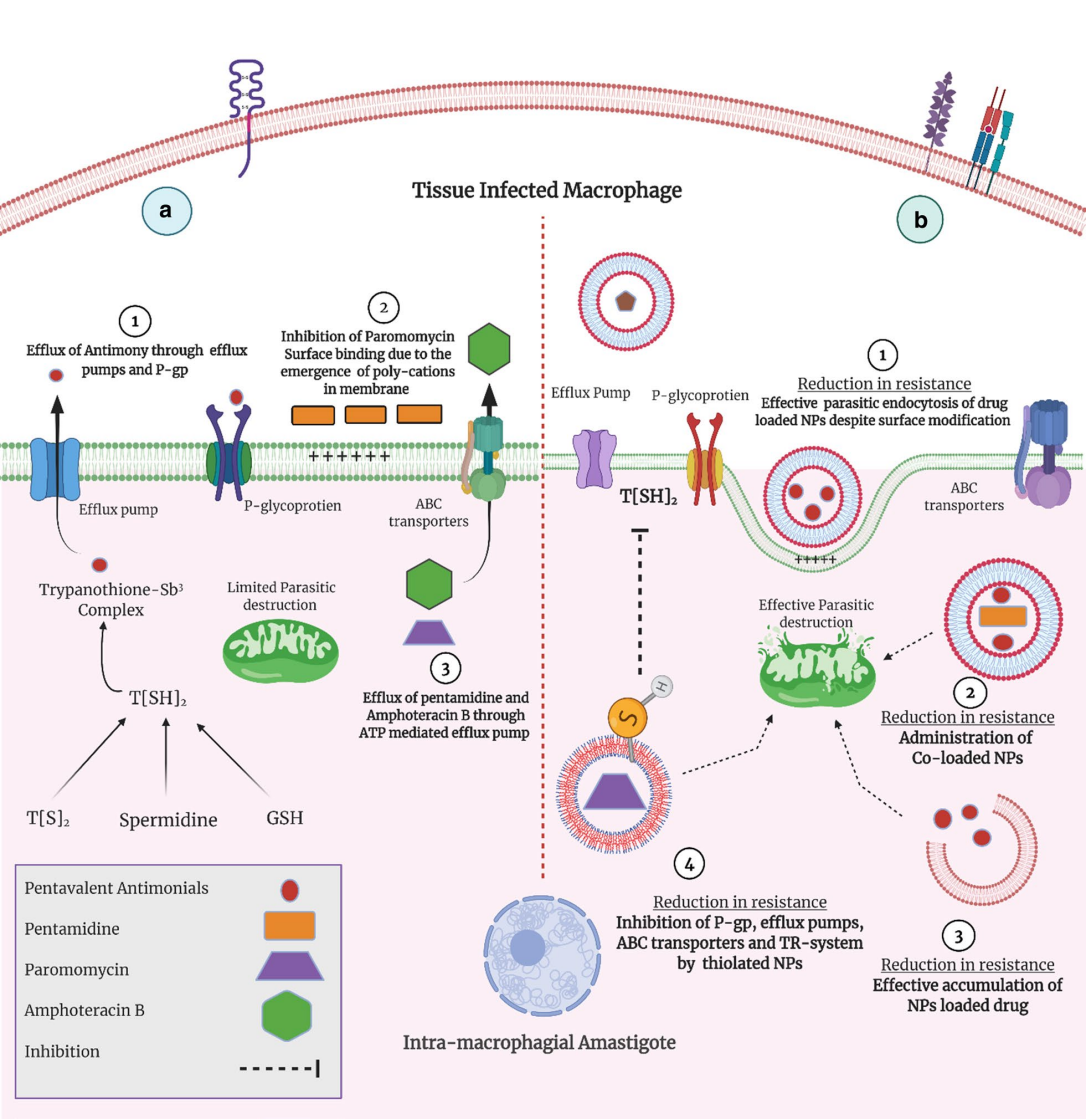
**a** Mechanisms of parasitic resistance against various anti-leishmanial drugs; [1] Drug expulsion through P-gp and TR-system mediated efflux pumps, [2] Reduced membrane binding due to repulsion between the drug and the parasitic plasma membrane, [3] Expulsion of drug through ATP-driven efflux pumps. **b** Ways, by which, nano-DDS curb the emerging resistance; [1] improved endocytosis of nano-DDS inside the parasite, [2] Co-loaded nano-DDS exhibits improved parasitic killing, [3] improved intramacrophage accumulation of nano-DDS, [4] Thiolated and other innovative nano-DDS inhibit P-gp efflux pumps and parasitic TR-system

There is no vaccine yet approved for Leishmaniasis prevention, but undergoing clinical trials [30]. Thus, the mainstay of treatment; for all forms of Leishmaniasis, relies on chemotherapeutic agents. Despite the extensive research on anti-leishmanial chemotherapy in the past 7 decades, satisfactory treatment has yet to be approved. Although, WHO has recommended the use of Meglumine Antimonate (MA) for all forms of Leishmaniasis, still it encourages the investigation of new anti-leishmanial agents because of multiple limitations. Nowadays, both of the above-mentioned cornerstones of leishmanial chemotherapy have gone resistant and not properly responding in terms of parasitic eradication [31]. In various regions of India, particularly in Bihar, numerous resistant cases of *L. donovani* have been reported. Consequently, numerous studies have been conducted and recommended the adoption of Amphotericin B (Amp-B) as an alternative first-line agent for resistant Kalazar (VL) cases [32]. Approval of Liposomal Amp-B (L-Amp-B), however, is presently being opted as a suitable alternative to antimonate therapy, particularly for VL. An unusual case of Amp-B resistance has been reported in India, according to the recently published case report [33]. Miltefosine (MFS) is the first oral anti-leishmanial agent approved by the Food and Drug Administration (FDA) for the management of CL, caused by *L. donovani,* and for some resistant cases of VL, it is merely, a ray of hope [34]. Despite the fact of its discovery, two MFS resistant cases have been reported in India [35]. The proposed mechanisms by which the leishmanial amastigote exhibit resistance against various agents has been graphically illustrated in Fig. 4a, b.

Two major mechanisms are involved in the emergence of resistant leishmanial strains, the ABC transporters (setup in the parasitic membrane) and thiol conjugated system (present in the cytosol). These ATP-binding cassette (ABC) transporters are also shown to minimize intra-parasitic drug accumulation accompanied by their rapid expulsion. ABCC3 transported is found to be associated with antimony resistance and these pumps work more efficiently to pump out thiol conjugated metal moieties. Pentamidine (PTM) resistance, evaluated in previous studies, was considered to be due to Pentamidine resistant protein-1 (PRP1) transporter [36]. In addition, due to various efflux pumps, P-glycoprotein (P-gp) and ATP mediated efflux pump, the drug residence time inside the parasite gets lowered which could ultimately lead to treatment failure. Thiol conjugation—Trypanosomatid Trypanothione reductase system (TR-system), also referred to as T[SH]_2_, which is merely an anti-oxidant system of the parasite also, unfortunately, plays a crucial role in neutralization and expulsion of drug [36, 37]. Moreover, the reason for Paromomycin (PMC) resistance is the reduced initial cationic drug binding with negatively charged leishmanial glycocalyx which would ultimately minimize the endocytosis as well as intra-parasitic accumulation of PMC. However, the detailed molecular mechanism of PMC resistance is yet to be discovered [38].

### Chemotherapeutic toxicity—hindrance in medication adherence

In addition to resistance emergence, MA has several other complications as well, such as large doses are required to meet up with the rapid parasitic clearance [39]. This possibly could lead to MA toxicity—renal failure, pancreatitis, and cardiomyopathy. Another reason for the decline in its use is its systemic toxicity. Sodium Stibogluconate (SSG), another pentavalent antimonate, has also been associated with systemic and local toxicity. Infected patients, undergoing SSG treatment, usually encounter pain at the site of injection as well as nausea and vomiting [40, 41].

Despite the effectiveness of Amphotericin-B (Amp-B) against fungal and leishmanial infections, its use has been restricted due to its systemic undesirable events and toxicity. Infusion-related adverse effects and nephrotoxicity are its major limiting parameters [42]. Pentamidine (PTM) therapy is often accompanied by problems such as painful necrotic injection site lesions, nephrotoxicity, and hypoglycemia; the main reasons for its poor adherence and associated lower cure rates [43, 44]. The discovery of anti-leishmanial activity of MFS is, merely, a novelty because it is the first oral anti-leishmanial drug for the treatment of Leishmaniasis [45, 46]. Although MFS is approved for oral treatment in Leishmaniasis, still some undesirable events such as gastrointestinal distress, hemolysis and nephrotoxicity are its major dose-limiting parameters and are the reason for poor patient compliance [47–49].

### Anti-leishmanials with meager GIT absorption

**Fig. 5:**
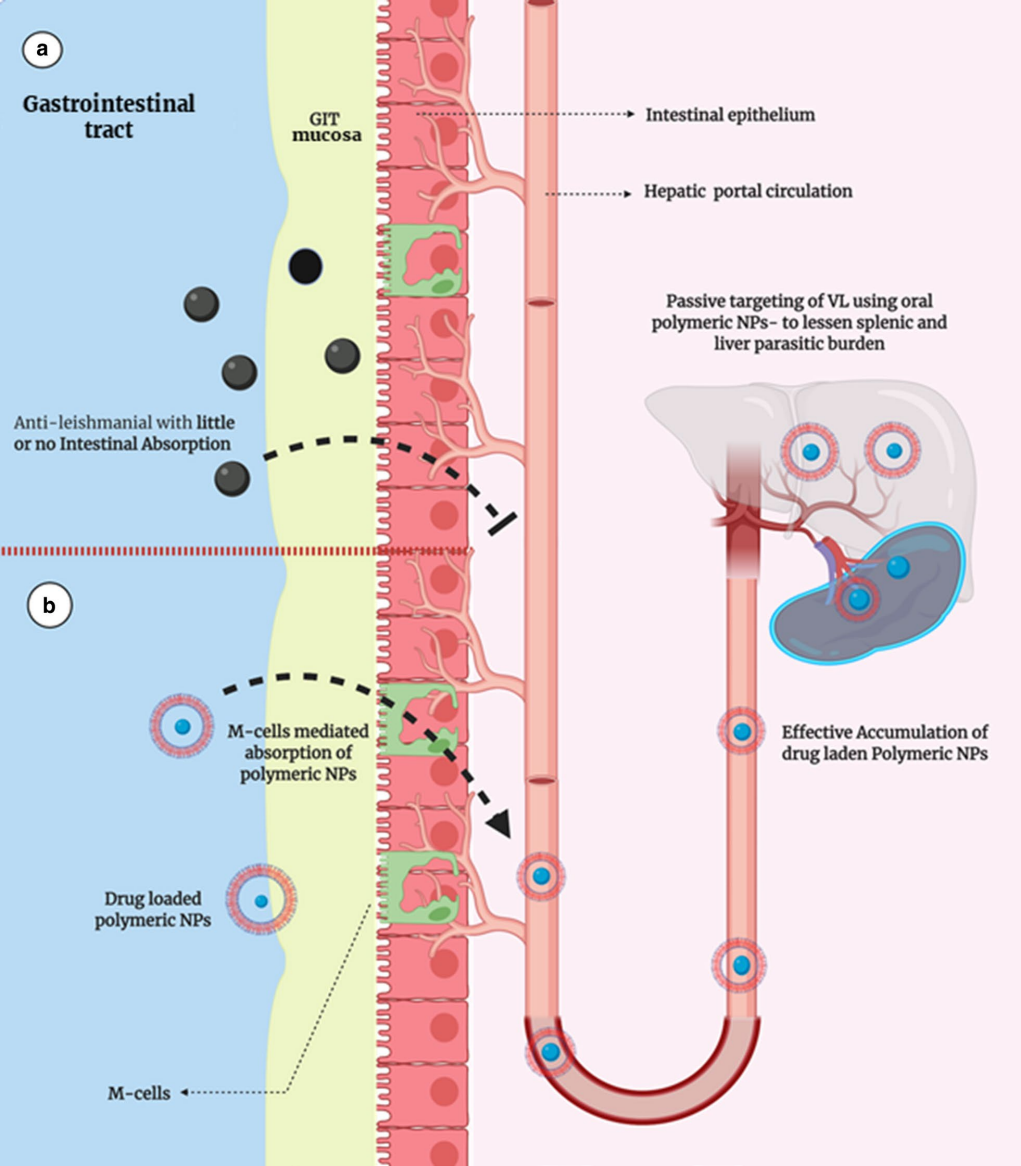
**a** Inadequate intestinal absorption of traditional anti-leishmanial drugs. **b** Improved M-cells mediated intestinal absorption, hepatic accumulation and oral bioavailability, hence, passive VL targeting could be achieved using anti-leishmanial nano-DDS

All 1st line anti-leishmanial drugs are injectable except MFS. Older anti-leishmanials; MA and SSG, are unable to cross the intestinal epithelial membrane, thus exhibit very poor oral absorption, illustrated in Fig. 5a, b. As a result, they are given as injectable and the preferred route of administration is intravenous and intra-lesioned. Amp-B, despite its good effectiveness against fungal and leishmanial infections, is administered intravenously and it eventually causes infusion-related side effects and nephrotoxicity [42]. Amp-B demonstrated passive diffusion for its intestinal absorption. However, due to its high molecular weight (924.08 Daltons), its intestinal passive diffusion was minimal. Moreover, it is also susceptible to damage caused by the acidic pH of the stomach [50, 51]. To tackle this challenging situation, several Amp-B nano-DDS have been designed (discussed in “Promising nano-DDS—capable for oral delivery of injectable anti-leishmanials” section). PTM is an aromatic diamidine available only in injectable form because of its stunted oral absorption. Chemically, it possesses two strong amide groups which is the major contributory factor towards its poor intestinal absorption [52, 53]. PMC, an aminoglycoside antibiotic, is a new treatment modality for Leishmaniasis and undergoing clinical studies. However, apart from its associated benefits, it also has limitations to be administered through parenteral, topical, or transdermal route only [54].

### Additional miscellaneous chemotherapy drawbacks

In the pursuance of a recommendation by WHO to use topical agents for uncomplicated CL, a few anti-leishmanials were fabricated to be applied topically to passively target the cutaneous parasites. This route is also beneficial for minimal systemic exposure and unnecessary drug metabolism [55]. Minimal cutaneous absorption of some anti-leishmanials is troublesome because the parasite resides in the deep cutaneous layer. To effectively treat CL, the drug must cross the epidermal barrier and internalize inside skin macrophages, but existing conventional dosage forms are unable to meet this condition [13]. Several other drugs possess leishmanial killing potential, but not enough to cause total parasite eradication, and are used as “off-label drugs”. Though they are safer drugs, however, to attain effective parasite eradication and disease remission, combined administration with 1st line anti-leishmanials is necessary. These agents include several anti-fungal azoles such as fluconazole and ketoconazole, rifampicin, stimaquine, and immunomodulators—Resiquimod (RMQ) and Imiquimod (IMQ) [40, 56]. The existing chemotherapy together with its associated obstacles is mentioned in Table 1.

**Table 1:** Anti-leishmanial chemotherapy and associated limitations

Drugs	FDA approval	Administration route	Possible associated challenges	FDA approved nano-formulation	References
Meglumine antimonate	Not approved	IV/IM/IL	Systemic adverse effects (Acute pancreatitis, Acute renal failure, hepatitis & cardiotoxicity) Resistance development^a^ Poor oral absorption	No	[31, 40, 41, 52]
Sodium stibogluconate	Not approved	IV/IM/IL	Systemic adverse events (Infusion-related toxicity (pain, nausea & Vomiting) with respiratory distress, vomiting) Resistance development^a^ Poor oral absorption	No	
Amphotericin B	Approved for VL caused by any species Particularly in Antimonials unresponsive cases	IV	Systemic adverse effects (Acute infusion-related toxicity & Nephrotoxicity) Resistance development^b^ Cost of therapy	AmBisome® (liposomes)	
Pentamidine (isethionate)	Not approved	IM/IV	Systemic adverse events (Nephrotoxicity & dysglycemia) Poor oral absorption	No	
Miltefosine	VL caused by *L. donovani* CL caused by *L. viannia*	Oral	Systemic adverse events (Gastrointestinal distress, elevated liver enzymes & renal toxicity)	No	
Paromomycin	Not Approved	Topical/IV	Poor oral absorption Variable response against CL Limited skin permeation	No	

^a^Almost 100% of the cases of VL in India are un-responsive to antimonial therapy

^b^A very few cases have been reported yet

### Futile vaccination candidates—another contest to confront Leishmaniasis

At present, myriad candidates of leishmanial vaccines are undergoing pre-clinical trials; live (genetically altered), live attenuated, killed parasitic vaccine, recombinant vaccine, recombinant protein, DNA vaccine, and antigen cocktail vaccine [57]. However, due to various obstacles, only a single vaccine candidate has been successful to reach the stage of human trials. Leish-111F has successfully completed phase-I and II clinical trials. Unluckily, it failed to exhibit a protective response in dogs as per the results of phase-III trials [57, 58]. The most probable barriers towards Leishmaniasis vaccine fabrications are marginally recoverable profit—as its heavy-budgeted program costing 300–800 million USD. Leishmania, being a disease endemic to under-developed and 3rd world countries, the vaccine industries presume to have minimal turnover and revenue generation. Inadequate effectiveness—because of minimal antigenic exposure to the body’s defending lymphocytes, of trial candidates is another obstacle in the way of vaccine commercialization. Furthermore, variable virulence of different leishmanial species and broad manifesting spectrum of the disease is also hampering immunization influx towards FDA approval [57].

## Strategies to overcome anti-leishmanials associated limitations

To confront the erstwhile discussed challenges accompanying the anti-leishmanial therapy and assure successful treatment of Leishmaniasis several strategies can opt. New drug development is one of the strategies used to discover, fabricate and optimize new and effective drugs. This is, however, a protracted (require 10–12 years on average) and high-budgeted approach. Besides, the risk of failure, the financial loss is also considered to be its limitations [59]. Despite these issues, scientists are using several biologically active substances as potential antileishmanial agents. Among them, edelfosine-an alkyl-phospholipid, has displayed valuable antileishmanial properties and is found more effective than MFS [60, 61]. The number of phenolic compounds has been assessed and found to possess pronounced in vivo and in vitro anti-leishmanial activity [62]. In the struggle to find a new treatment option, the anti-leishmanial activity of calcium channel blockers, fendiline, and lidoflazine, was reported but due to unsatisfactory activity against intracellular amastigote research was unable to be continued [63]. The discovery of IMQ is however one of the scientist’s successful efforts. As some of its analogs possess strong leishmanicidal properties [64]. The second strategy is the development of new derivatives of existing anti-leishmanial drugs for the reason to alter the pharmacokinetic (ADME) parameters, toxicity profile, and enhancing their potency. By using this technique, derivatives can be designed that have effectiveness against resistant cases. One such attempt was made, to modify the PTM using boronic acid to target the leishmanial membrane lipophosphoglycan. But the results of the study were not satisfactory [65]. In another meaningful attempt, MA with a low polymerized state has been developed which exhibited considerable oral absorption [66]. Primaquine (PMQ), having reported anti-leishmanial activity, was chemically modified by the introduction of imidazolidin-4-one and ferrocene to form peptidomimetic and organometallic derivatives of PMQ, respectively. In this study, the anti-leishmanial activity of the above-mentioned derivatives against intracellular amastigotes of *L. infantum* was reported to be less than the parent compound and the positive control, the MFS [67].

### Nanotechnology—a revolutionary perspective towards medical advancement

**Fig. 6:**
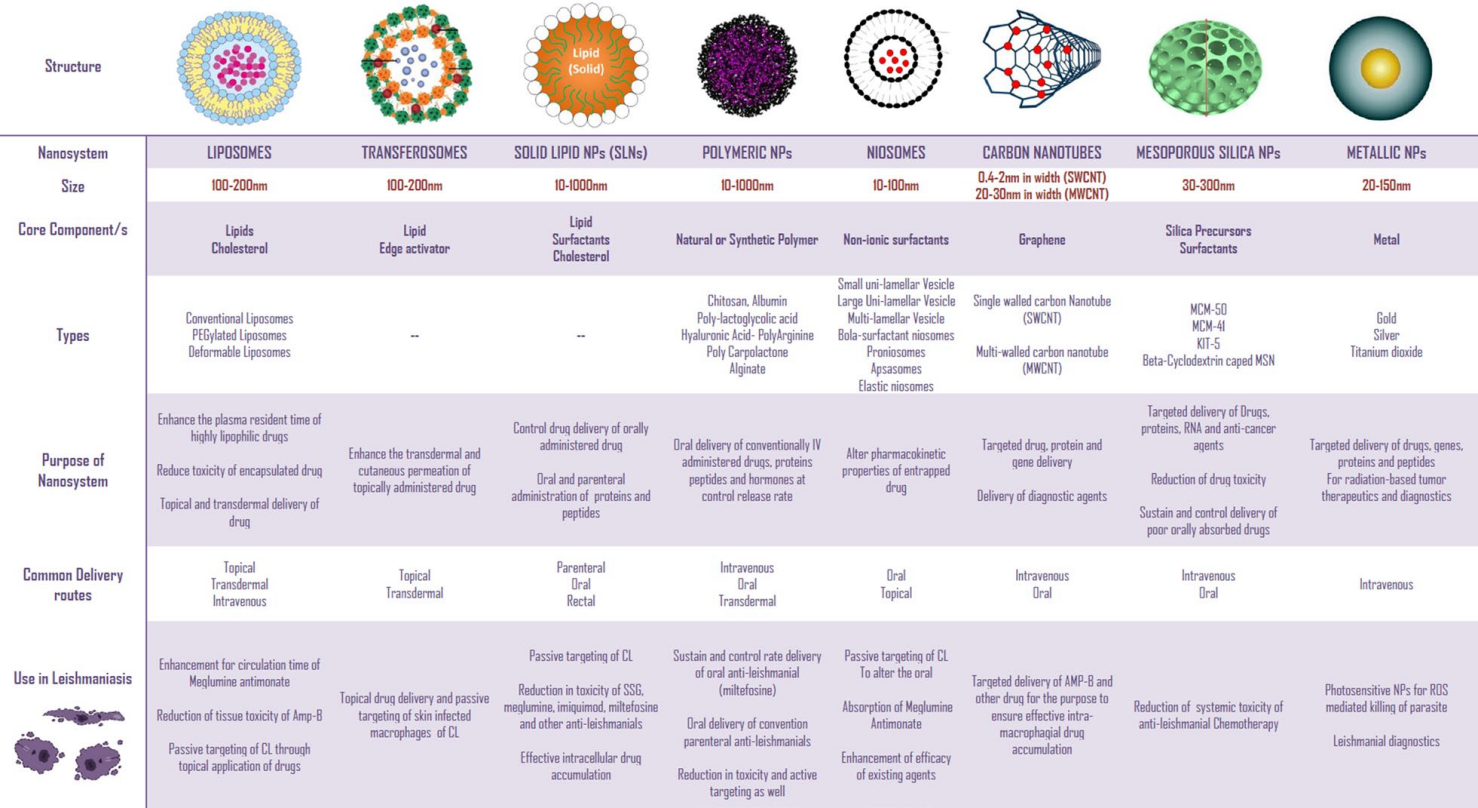
Nanoparticles employed to grapple anti-leishmanials associated drawbacks

The preferable option to hasten the limitations of anti-leishmanial chemotherapeutic and immunogenic agents is their nano-optimization. It is also be referred to as nano-medicine which is defined as the use of nano-technology for the sake of increasing the solubility, modifying the pharmacokinetics, reducing toxicity, and enhancing tissue targeting in contrast to the conventional options [68, 69]. Thus, with the employment of nano-DDS, the oral bioavailability of inadequately absorbed drugs will improve along with the impartment of sustained and controlled release behavior [70, 71]. As previously mentioned, the conventional drug delivery systems are not capable of targeted delivery. Nanotechnology-based DDS have also resolved this contest as they can easily be modified using surface tuning with certain biological moieties. Those surface-attached moieties act as a guide towards the target tissue. This targeting approach is referred to as “active targeting” [72]. Another important aspect is their ability to achieve enhanced therapeutic efficacy in a shorter treatment duration, contrary to the conventional drug delivery systems [73]. Generally, in context to Leishmaniasis, the nano-DDS of conventional anti-leishmanials grapple with several limitations inclusively their resistance, toxicity, infection re-emergence, and pharmacokinetics restrictions [74, 75]. These findings are extensively discussed in next “Nanosystem with upgraded efficacy for curtailment of chemotherapeutic toxicity”; “Promising nano-DDS—capable for oral delivery of injectable anti-leishmanials”; “Targeted delivery of anti-leishmanials—unfettered access to intramacrophage parasite”; “Nanosystem based modalities to curb parasitic resistance” and “Distinctive nano-DDS for Leishmaniasis” sections of the review. Nanotechnology, in addition to drug delivery, has also shown its worth in the immunization domain. Nanocarriers offer safe and efficient trafficking of immunogenic proteins to the Antigen-presenting cells (APCs). Furthermore, nano-vaccines are also much more stable than convention vaccines even inside the blood circulation [76]. This immunization approach has also unfolded a new research horizon—an oral vaccine delivery. Numerous nano-adjuvants, under pre-clinical and clinical investigation, are discussed in detail in “Leishmanial nano-vaccines—an innovative approach towards immunization” section. Metallic Nanoparticles (NPs), in particular Gold NPs (AuNPs), dendrimers, and Quantum dots (QDs) also retain potential diagnostic applications that have been evaluated for leishmanial parasite spotting as well [77]. Despite the lack of reasonable outcomes i.e., improper selectivity, sensitivity, and erroneous (false-negative) results, research on leishmanial nano-diagnostics is still advancing. The use of nano-repellants, nano-insecticides, and biological nanoparticles for the purpose to control the vector activity is another obscure field that requires considerable attention, in particular, for leishmanial endemic countries [78]. Nano-theranostics, another forward-looking utilization of NPs for simultaneous diagnosis and treatment, are also under pre-clinical evaluation. This forthcoming modus operandi will greatly influence the treatment outcomes and recovery time, positively [77]. Among them, AmBisome®, the L-Amp-B, is the only approved anti-leishmanial nano-DDS based product, providing a non-toxic treatment for VL [79]. In this review, the nano-DDS, designed in last decades for trumping leishmanial treatment, immunization and prevention have been discussed in a comprehensible and critical manner, particularly in terms of in vivo assays. Frequently designed nano-DDS for leishmaniasis, their structure, composition, features, benefits and uses are illustrated in Fig. 6 and discussed comprehensively.

#### Lipid-based nano-DDS

Liposomes (LPS), artificially fabricated, spherical, vesicular nanosystem, usually constructed using natural or synthetic lipid moieties and the cholesterol, merely, to strengthen and stabilize the designed lipid bilayer. The dual incorporation of hydrophilic and lipophilic drugs in LPS make them the most suitable and worthy nanocarriers for drug delivery through a variety of routes i.e. parenteral, topical, oral, etc. [80]. LPS are also beneficial in terms of passive targeting to various APCs and tissue macrophages. Polyethylene glycol (PEG)-coated LPS are more stable and have considerably improved half-life (plasma circulation time) as compared to the conventional LPS. Another innovative strategy to prolong the circulation time of NPs is to fasten CD47 protein that appears to interact with SPIR-α macrophage receptors. This interaction ultimately reduces the phagocytosis rate of NPs, hence plasma circulation time lifts [81]. Solid lipid NPs (SLNs), have the potential to entrap a vast variety of drugs, proteins, and other medicinal entities. These NPs are considerably stable owing to surfactant covering around them [82]. Due to their small size and lipid nature, they prevents the toxicity incidence of anti-leishmanial agents by limiting their release and exposure [83]. This modified lipid nano-DDS has resolved the stability issues linked with LPS, but experienced burst release of the entrapped drugs. This has hampered the SLNs usefulness towards nanomedicine [84]. Surface modification of SLNs can be effective in controlling the drug release rate [85]. Utilization of a mixture of unstructured solid and liquid lipids along with surfactants in a ratio of 1.5–5% (weight/volume) has minimized the undesirable burst release of SLNs. Nanostructured lipid carriers (NLCs), in addition, are highly stable and multi-purpose i.e. used for oral as well as dermal delivery. A valuable study has reported the NLCs potential to inhibit intestinal P-gp efflux pumps [86]. Flexible phospholipid-based nanocarriers, the Transfersomes (TFS), have been studied for passive targeting of CL [87]. These nano-DDS possess surged cutaneous penetration as proven by confocal laser scanning microscopy (CLSM) of dye-loaded TFS in numerous studies [88, 89]. In reality, this lipid structured nano-DDS was extensively investigated and utilized for the usefulness and effectiveness in leishmanial nanomedicine.

#### Biodegradable polymeric nano-DDS

Polymeric NPs facilitates in traversing biological barriers and membranes. The oral absorption is also facilitated through Microfold (M)-cells in the intestinal peyer’s patch [90]. The provision of pH stability is an additional benefit for pH-sensitive drugs. Furthermore, drug release in a sustained and controlled fashion, the elevation of bioavailability, efficacy improvement, tissue targeting, and adequate cellular uptake are all the paybacks of bio-polymeric nanocarriers. Among various biodegradable polymers, poly-lacto-glycolic-acid (PLGA) is the most successfully employed polymer. After cellular hydrolysis, PLGA is catabolized into lactic acid and glycolic acid. The human body effectively metabolized these monomers; thus, it is non-toxic. PLGA is an FDA-approved polymer to be utilized in commercialized nanomedicine [91]. Another attractive aspect is the easily attainable surface modification of PLGA-NPs, to impart selectively victimize cells, tumors, and infectious microbes [92, 93]. Poly-caprolactone (PCL), another hydrolyzable bio-polymer, is also often employed to synthesize polymeric nanosystem with better release properties, intestinal absorption, and selective targeting. Moreover, it has been utilized in an attempt to deliver insulin through the oral route. Hence, it provides extra-protection to proteins, peptides, and nucleic acids from the harsh gastrointestinal tract (GIT) atmosphere [94, 95]. Carbohydrate-based natural polymer, chitosan, is also valuable because it is associated with multiple benefits irrespective of its biocompatibility. Proteinaceous substances like cyclosporin A and insulin are easily delivered with adequate protection. Intrinsic anti-biological and anti-oxidant effects of chitosan strengthening the chitosan use as a polymer for nano-DDS [96]. Poly-lactic acid, gelatin, and dextran are other bio-polymer having applications in nanomedicine [94].

#### Additional miscellaneous nano-DDS

Another appropriate nano-carrier system—niosomes which are composed of non-ionic surfactants and lipids is capable to enhance the intestinal permeation of BCS class-II drugs. Moreover, these nano-formulations are also efficient in protein and vaccine delivery through the oral route. Provision of sustained and controlled release of entrapped drug and transdermal delivery of agents is also its beneficial features [97]. Mesoporous silica NPs (MSNs) can also be employed for efficient oral delivery of poor absorbable agents at sustained and controlled rate. These inorganic nano-DDS have a large surface area and pore volume, which ensures high loading efficiency [98]. Carbon nanotubes (CNTs) are the carbon-based nanosystem having condense benzene molecules spun to form a tubular structure having a length of 0.25–5 μm in length and 0.4–2 μm in diameter (depending on that it is single-walled or multi-walled). They are hydrophobic and bio-toxic, and to make their utilization for nanomedicine they should be functionalized [99]. Among metallic NPs, Silver NPs (AgNPs), Titanium oxide (TiO_2_NPs), and AuNPs display valuable significance for leishmanial nanomedicine, particularly for the development of phyto-nano-DDS; discussed in “Distinctive nano-DDS for Leishmaniasis” section.

## Intracellular trafficking of nano-DDS—major mechanisms and influential factors

**Fig. 7:**
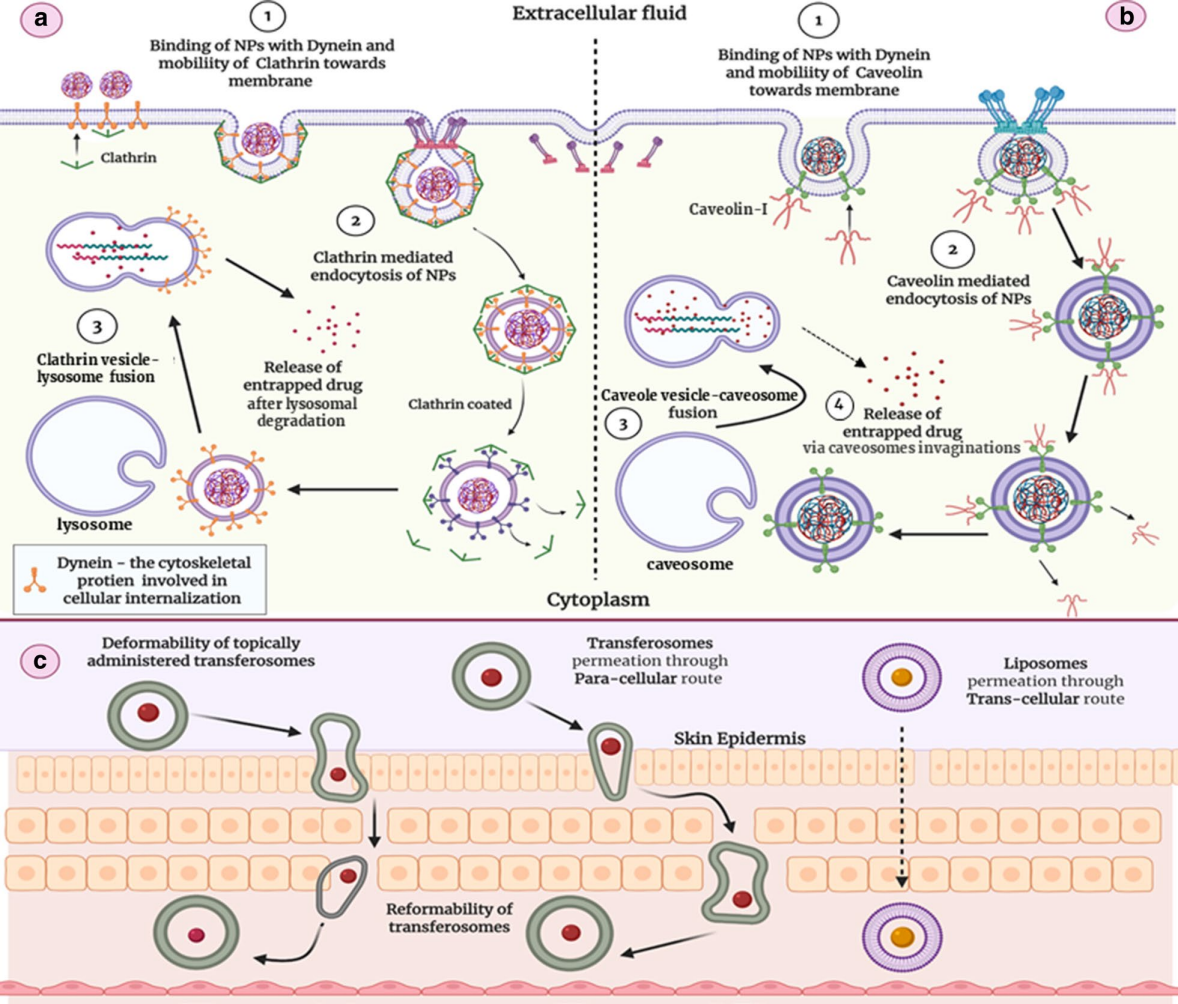
**a** Representing the Clathrin-mediated endocytosis for NP internalization. **b** Caveolin-mediated endocytosis of NPs. **c** Skin penetration mechanisms of Transferosomes and Liposomes

An outer covering of animal cells—the plasma membrane, is composed of a phospholipid bilayer and the embedded transporting and structural proteins. The arrangement of lipid bilayer permits only small molecules to traverse the membrane. Larger molecules (size range > 1 μm), however, experience intracellular mobility through “phagocytosis” [100]. Nano-DDS are capable of crossing biological barriers owing to their size in the nanometer range [101]. The NPs having a size below 200 nm usually undergo pinocytosis (endocytosis) viz*.* caveolin-mediated endocytosis and clathrin-mediated endocytosis [102]. The detailed mechanism for NPs internalization is illustrated pictorially in Fig. 7a, b. There is another pinocytotic pathway termed “clathrin-caveolae independent endocytosis” and in nanomedicine, it is not very much familiar. Folate labeled chemotherapeutic nano-DDS usually select this pathway to reach the target tumor [103, 104].

Clathrin is a cellular membrane protein responsible for vesicle construction, known as “Clathrin cage”, that conducts intracellular mobility of selective extracellular matters [105]. Clathrin-mediated endocytosis is a vital cellular internalization phenomenon by which cells usually uptake plasma components i.e., cholesterol, through Low-density lipoproteins (LDL) receptors and iron via Transferrin receptor [106, 107]. The major steps of Clathrin-mediated endocytosis include—binding of a ligand with the receptor at the plasma membrane, the formation of ligand-receptor complex, mobilization of the complex at the clathrin-rich portion of the membrane, and ultimately the formation of clathrin layered vesicle. Furthermore, that vesicle fuse with cytosolic lysosomes followed by the release of entrapped drug [108]. Generally, most of the lipid-based nano-DDS (neutral and positively charged) opt for clathrin-mediated endocytosis as a mode of cellular entry so does the PLGA NPs and MSNs [109, 110]. Polylactide NPs and plus-charged chitosan NPs also attain cellular access in this way [109]. There is a profound impact of surface coating over the NPs intracellular trafficking. The surface coated, modified, and labeled NPs undergo rapid and targeted internalization through Clathrin-based endocytosis [103]. In nanomedicine, caveolin-mediated endocytosis is preferred owing to the by-passing of lysosomal fusion. Caveolin-mediated endocytosis involves the formation of flask-like pits in the membrane having the size of 20–100 nm through the utilization of immobile lipid portions of the plasma membrane—the caveolin-1 [111]. Contrary to clathrin-mediated endocytosis, the NPs interaction with caveole results in formation of caveole-I vesicles which intracellularly fuse with larger caveolin-I based cytoplasmic organelle, having neutral pH, termed as “caveosomes or multi-caveolar complex” [112], as illustrated in Fig. 7b. After few hours of residence, the static caveosomes, entrapping NPs, became dynamic and start to fuse with smooth endoplasmic reticulum through membrane invaginations. Finally, the NPs shifted into caveole free membrane vesicles and released in the cytoplasm without any damage. Fortunately, the content entrapped inside caveolae does not undergo pH-induced degradation due to the neutral environment of caveosomes [113, 114]. Thus, this route is highly valuable for the safer delivery of nanomedicine [115]. Caveolin-mediated endocytosis is a lengthy internalization mechanism as compared to clathrin-mediated endocytosis but ensures safer intracellular delivery of NPs, as lysosomal fusion (Fig. 7a) of membrane pinched off vesicles is replaced with caveosomes fusion (Fig. 7b). Some insightful studies have evaluated that negatively charged nanomedicine utilized this pathway to move inside the cell. Thus, by modifying the NPs surface with negatively charged substances, this non-degradable pathway can be attained. This would ensure the safer and protected delivery of medicated agents [109]. As reported earlier, negatively charged PEGylated LPS were more effective than neutral and positively charged ones. Doxil®—Doxorubicin loaded negatively charged PEG covered LPS, elect endocytosis through caveolae formation [116]. Polymeric micelles with anionic core, Abraxane®, negatively charged QDs, and Polysiloxane NPs take advantage of this pathway as well [109, 117–119]. NPs cellular internalization pathway can be probed by various analytic and imaging techniques including confocal fluorescence microscopy, transmission electron microscopy, atomic force microscopy, photoacoustic microscopy light scattering and scanning electron microscopy. Apart from microscopic assessments flow-cytometry, surface enhanced Raman scattering and X-ray adsorption near-edge spectroscopy are used [104].

Their cellular internalization and trafficking, however, can be manipulated by modifying its morphology (size and shape) and surface structure [120]. However, the maximal cellular uptake was observed with particles having a size of 50 nm [121]. Caveolin and clathrin-mediated endocytosis is the predominant mechanism of intracellular trafficking for NPs having a size of less than 500 nm. The mechanism shift is encountered as the size gets increased. Thus, the particles with a size greater than 1000 nm undergo cellular internalization through phagocytosis [121]. With respect to shape, spherical NPs undergo significant cellular accumulation as compared to other shapes as this factor also appears to have a pivotal role in NPs uptake [122]. However, there is the availability of the data showing entirely different results, for instance, the rod-shaped NPs have better internalization than spherical-shaped nano-DDS [123]. Elasticity, the deformability, of NPs is another critical property that influences their absorption and internalization. Several studies have provided evidence that firm NPs have lesser cellular uptake than elastic NPs [103]. QDs also undergo macro-pinocytosis for cellular internalization [124]. Charged bearing NPs also have a critical impact on the cell internalization pathway. As discussed earlier, negatively charged NPs preferred caveolin-mediated endocytosis—a lysosome bypassing route [109]. Focusing on cutaneous delivery of nano-systems, lipid-based nano-formulation is usually preferred, because of their ability to perforate through tight cutaneous junction efficiently. TFS, owing to their deformable nature, is considered as the most appropriate and recent carrier to targeted drug delivery to cutaneous macrophages—as they plump themselves to attain deeper cutaneous penetration using a transcellular route [125–127], and it is illustrated in Fig. 7c.

This evidence-based discussion, about the influencing factors and the route opted by the NPs for cellular uptake and targeting, is fundamental for understanding the role of nano-DDS in tackling the drawbacks of conventional anti-leishmanials. Ending up with a general discussion, the next portion of the review contains data and discussion about the nano-DDS approach for resolution of erstwhile discussed obstacles, encountered during the management of Leishmaniasis.

## Nanosystem with upgraded efficacy for curtailment of chemotherapeutic toxicity

As mentioned earlier, nano-DDS are comparatively safer than conventional drugs. Far too many LPS nano-DDS have been designed and emerged with noteworthy results. In 1997, L-Amp-B got FDA approval and ranked as the only FDA-approved nano-formulation currently under clinical use for the treatment of Leishmaniasis [79, 128, 129]. Interestingly, the L-Amp-B was found to significantly minimize Amp-B deoxycholate nephrotoxicity with retaining comparable effectiveness [130]. MA loaded plain LPS and PEGylated LPS have fortunately undergone a high depression in drug toxicity particularly in pancreatitis and cardiomyopathy, possibly due to minimal exposure of MA with normal body tissues. Pharmacokinetic and in vivo anti-leishmanial activity evaluation was also carried out in *L. infantum* infected male mongrel dogs and Swiss mice VL models. Furthermore, in comparison to plain MA-LPS, MA-PEGylated-LPS retain valuable pharmacokinetic parameters—improved half-life, better accumulation in target tissues—spleen and liver, and maximal eradiation of the parasites. Higher plasma MA levels coupled with minimal tissue toxicity were encountered with MA-PEGylated-LPS. However, even after 14 days of treatment complete eradication of parasitemia was unable to be achieved with MA-PEGylated-LPS. These designed PEGylated LPS of MA can opt as a suitable option to sustain drug tolerability and efficacy, for which, more extensive in vivo studies would be required [131]. The incorporation of MFS inside lipid based NPs (composed of oleic acid, cotton seed oil, phosphatidylcholine and cholesterol with size range of 100 nm or less and highly efficient encapsulation) has found a significant decline in MFS-induced hemolysis. The hemolytic activity of the designed nano-DDS was compared with the free drug, at two different concentrations. It has been observed that percent hemolysis occurred with 175 μM MFS is 5% for nano-formulation and 73% with the plain drug. Moreover, 350 μM MFS in the designed formulation and free drug showed 13% and 100% hemolysis, respectively. All the experiments in this study were conducted in vitro, hence, there is a need to evaluate the tolerability and anti-leishmanial efficacy of MFS lipid nanoparticles in the in vivo setting as well [49]. These nanocarriers were examined majorly by in vitro hemolytic assay, in vitro macrophage toxicity, GIT toxicity in the rats, and leishmanicidal assay using the BALB/c mice model. Hemolysis (%) was reduced from 91% (for MFS solution) to 8%. Values of CC_50_ also improved significantly to 237.4 μg/ml, however, with MFS solution it was 39.60 μg/ml. Furthermore, the results of animal studies were revealed to minimize MFS induced GIT epithelial and mucosal damage along with the significant reduction in leishmanial lesion size in BALB/c mice, in contrast to the simple drug solution. However, more advanced in vivo studies—lesion and organ parasitic burden reduction, are the pre-requisite for further progressing of MFS-NLCs towards human studies [132]. IMQ, an agent with limited anti-leishmanial activity, proved to possess efficacy against CL when applied topically in form of 5% w/w cream. However, cutaneous reactions and psoriatic like condition following halt its use [133]. IMQ-loaded SLNs were evaluated for their toxicity using MTT assay. In MTT assay, human epithelial cells; HaCaT cells, were used. The results of the MTT assay showed the A (Absorbance value) as 0.555 of IMQ-SLNs at 490 nm, which was lower than the free drug i.e. 0.563. These results were suggesting higher IC_50_ value and better safety profile of the designed nano-DDS than the plain drug. However, this study has multiple limitations including very few characterization studies, showing A value instead of IC50 values and a lack of anti-leishmanial assay [134]. As in clinical trials, infection recurrence has been reported owing to IMQ lessen cutaneous permeation [56]. Thus, the fabrication of IMQ nano-DDS possibly potentiate its anti-leishmanial efficacy and ameliorate cutaneous toxicity [135]. Several biodegradable polymers are recently used for the synthesis of these nano-DDS including—synthetic polymers i.e. PLGA, PCL, and natural polymers i.e. Chitosan, Albumin, alginate, and the list goes on [136]. Likewise, lipid nano-DDS, polymeric NPs can also be used in the toxicity reduction of the entrapped drugs. They facilitate passive drug targeting and prevent unnecessary exposure of the drug to normal body tissues and organs [137]. Chitosan-chondroitin sulfate NPs loaded with Amp-B (Amp-B-CCs-NPs) was found to have valuable surge in activity against laboratory parasite (*L. amazonensis and L. chagasi*), followed by enhanced intra-macrophage accumulation as compared to the plain drug. Polymer utilized for this nanosystem have also imparted a sustained release pattern of Amp-B. Another valuable characterization has been conducted to estimate the NPs internalization inside macrophages. The researchers have designed fluorescein isothiocyanate (FTIC) labelled chitosan-chondroitin sulfate NPs and quantify their macrophage internalization using CLSM mainly due to optimal particle size of 136 ± 11 nm. This study was an indicator of higher buildup of Amp-B-Chitosan-chondroitin sulfate NPs in infected macrophages as it is directly related to its improved leishmanicidal activity, in comparison to the plain Amp-B. The uptake was coupled with reduction in Amp-B cellular toxicity (CC_50_ = 9 ± 0 μg/ml), hemolysis (RBC_50_ = 240 ± 33 μg/ml) along with efficacy improvement—IC_50_ = 1 ± 0 μg/ml and 0.1 ± 0.1 μg/ml against *L. amazonensis and L. chagasi*, respectively. However, this research also lacks an in vivo anti-leishmanial assay, which is crucial to confirm its utilization as an anti-leishmanial nano-DDS [138]. With a similar aspect, alginate NPs enclosing MFS was designed for the purpose to reduce MFS-associated hemolytic anemia. In this study, the cytotoxicity of designed nanomedicine was evaluated against RBCs. The *G. mellonella* larvae were also employed for toxicity detection. Having MFS concentration of 35 μg/ml, the drug solution caused 50% hemolysis in contrast to the MFS-alginate-NPs, which had no impact on RBCs morphology and destruction rate. In addition, the plain drug showed 33% mortality of larvae at 50 mg/kg for 5 days. Luckily, only 5% larval mortality was observed with nanosystem delivering 200 mg/kg of MFS. The results of the study were highly supportive of the fact that MFS hemolysis and cytotoxicity showed considerable reduction due to MFS incorporation in the nanosystem. These nano-DDS were evaluated only for their anti-fungal potential against clinical strains of *C. albicans.* It is suggestive to conduct anti-leishmanial assay both in vitro and in vivo Leishmania-infected BALB/c mice for the purpose to assess leishmanicidal potential of MFS-alginate NPs. [139]. Several polymeric nanocarriers being employed for Leishmaniasis are stated in Table 2. Possibly, these nanocarriers prevent API from harsh environment. Polymeric coat imparts drug release pattern at control rate and gratuitous interaction of drug with healthy tissues mitigates. After all this discussion, we can conclude that polymeric NPs are promising instruments for anti-leishmanial delivery, ensuring significant reduction in their toxicity profile. Albumin NPs also have potential to improve safety and efficacy of entrapped agents. Recently, MA-albumin NPs were designed and evaluated for their cytotoxicity and efficacy parameters. The engineered nanosystem was found to greatly reduce IC_50_ values against *L. major* promastigotes along with valuable increment of CC_50_ value against J774 macrophages, using MTT assay. The IC_50_ and CC_50_ values of MA-Albumin NPs were 8.35 ± 0.92 μg/ml and 309.7 ± 2.49 μg/ml, correspondingly. However, plain MA possessed IC_50_ and CC_50_ values of 26.67 ± 1.42 μg/ml and 112.5 ± 2.05 μg/ml, which were significantly different i.e. less effective and more toxic, from erstwhile mentioned values of the designed MA-Albumin NPs [140]. Another research reported by Casa et al. [141] has claimed the reduction in Amp-B toxicity by designing Amp-B loaded bovine serum albumin NPs (Amp-B-BSA-NPs). However, this study had multiple limitations, as they assessed toxicity only in laboratory J774.A1 macrophages and evaluated in vivo anti-leishmanial response only by estimating lesion size reduction. These results could be much better if parasitic eradication would be determined by estimating parasite load in the lesion as well as in the spleen and liver. However, due to supporting results of macrophage cytotoxicity assay, with 70-fold increased CC_50_ in comparison to plain Amp-B, and in vitro anti-leishmanial activity against *L. amazonensis* infected BALB/c mice, this candidate could be of interest for advance in vivo assessment for its claimed enhanced efficacy and reduced toxicity [141]. These properties are, however, not only limited to lipid or polymeric based nanocarriers. Several other nano-DDS have been synthesized to curtail anti-leishmanial unwanted and toxic outcomes. For instance, fabrication of Amp-B-loaded functionalized—CNTs (f-CNTs) were fruitful in downgrading the Amp-B associated hepatic and renal damage [142]. Toxicity studies were conducted in vitro and in vivo, using MTT assay and BALB/c mice models, respectively. Briefly, MTT assay compared the CC_50_ of plain Amp-B, f-CNTs, and Amp-B-f-CNTs. Amp-B-f-CNTs was found to be less toxic than plain Amp-B. Moreover, the designed Amp-B loaded f-CNTs were also non-toxic to the liver and kidneys as none of the liver or kidney destructive biological markers were elevated followed 5 days intra-peritoneal administration. Treatment with Amp-B-f-CNTs in *L. donovani* infected Syrian golden hamsters has exhibited better efficacy with plain Amp-B in terms of spleen weight reduction, parasitemia reduction, and inhibition of parasite replication. In order to advance this candidate towards clinical trials, its efficacy and safety should be compared pre-clinically with L-Amp-B, as Amp-B-f-CNTs are superior due to low production cost and stable nature [142]. Likewise, MSNs are also capable of a safer supply of drugs with minimal exposure to normal body tissues and organs. Findings have claimed that loading PTM-MSNs have significantly minimized its major dose-limiting adverse event—the nephrotoxicity, mainly due to incorporation of drug release at control rate. Inadequate loading of PTM isethionate inside NPs was resolved by simply de-salting it [143].

**Table 2:** Promising leishmanicidal-laden Nano-DDS with a potential of toxicity reduction

Type of nanosystem	Nanosystem engineered	Loaded anti-leishmanial drug	Characterization	Leishmanial strain	Animal model	Major results	References
Lipid-based nanocarriers	PEGylated Liposomes	Amphotericin-B	Human studies	*–*	–	Improved patient tolerance Reduction in infusion-related and systemic toxicity Improvement of the pharmacokinetic profile of Amp-B	[144]
	PEGylated Liposomes	Meglumine Antimonate	In vivo	*L. infantum*	Mongrel dogs and BALB/c mice	Reduction in systemic toxicity, improved circulation time, and significant parasitic burden reduction in liver and spleen	[131]
	Solid Lipid NPs	Imiquimod	In vitro	*–*	–	Reduced cytotoxicity evaluated in MTT assay using HaCaT cells	[134]
Polymeric nanocarriers	Chitosan-Chondroitin sulfate NPs	Amp-B	In vitro	*L. amazonensis* *L. chagasi*	–	Enhanced activity against *L. amazonensis* with a considerable surge in intramacrophage uptake 90% parasitic killing in infected macrophages	[138]
	Bovine serum albumin NPs	Amp-B	In vitro + In vivo	*L. amazonensis*	BALB/c mice	Amp-B-BSA-NPs, with a mean size of 173 ± 5 nm, was found to exhibit a higher CC_50_ value (70-fold than plain Amp-B), lower IC_50_ to that of the plain drug, and reduce lesion size after 2 weeks of treatment	[141]
	Albumin NPs	Meglumine Antimonate	In vitro	*L. major*	–	Drug loaded Albumin NPs were experimented with and found to have lower IC_50_ as compared to the plain drug accompanied with the highest CC_50_	[140]
	Alginate NPs	Miltefosine	In vitro + In vivo	*–*	*G. mellonella* Larvae	MFS loaded Alginate NPs observed with no hemolytic and cytotoxic properties	[139]
Graphene-based nanocarriers	Carbon Nanotubes	Amphotericin-B	In vitro + in vivo	*L. donovani*	BALB/c mice	No sign of any sort of damage was observed in BALB/c mice models particularly the absence of renal and hepatic toxicity	[142]
Silica-based nanocarriers	Mesoporous Silica NPs	Pentamidine	In vitro	*–*	–	Drug loaded MSNs exhibit control release of encapsulated drug and minimal nephrotoxicity associated with pentamidine parenteral administration	[143]

## Promising nano-DDS—capable for oral delivery of injectable anti-leishmanials

**Fig. 8:**
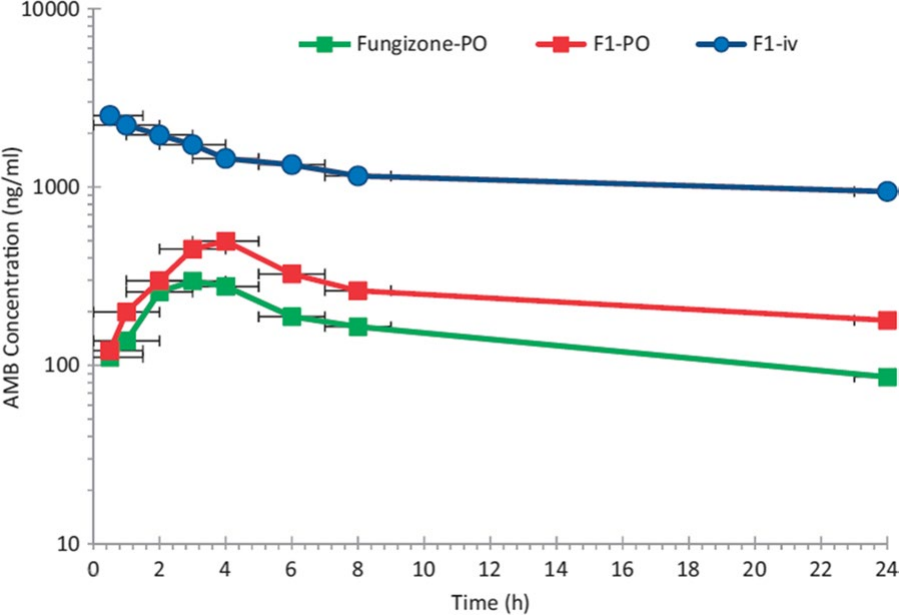
Graph plotted against mean plasma concentration (ng/ml) of Amp-B and time (h) clearly demonstrating improved intestinal absorption and bioavailability of Amp-B followed by oral administration (Dose = 10 mg/kg) of Amp-B-PLGA NPs (F1-PO) in comparison to Fungizone®-PO in healthy rats [147]

**Fig. 9:**
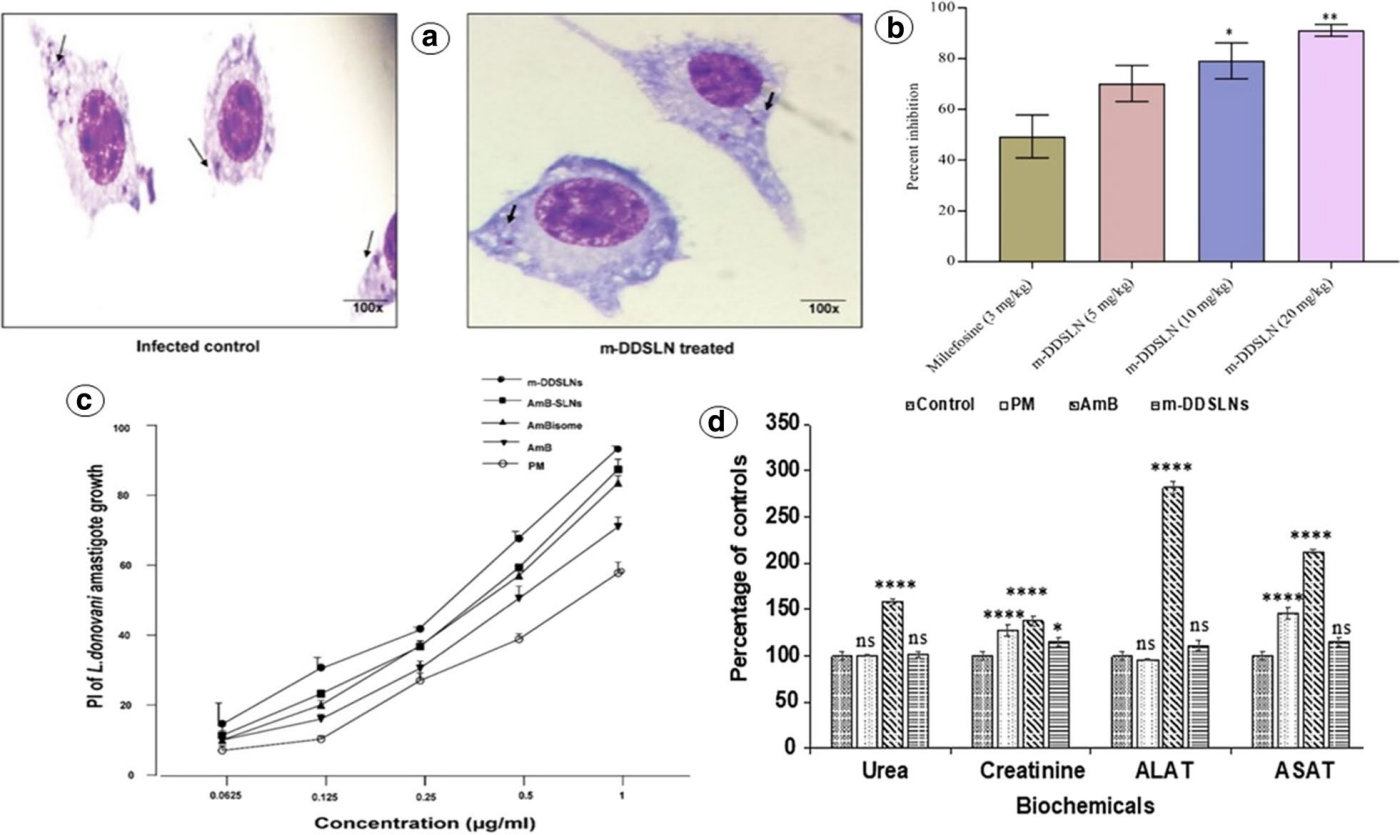
**a** Microphotographs of pre-treatment infected macrophage (left) and reduce intra-macrophagial parasitic burden post treatment with the surface modified drug loaded SLNs (m-DDSLNs) [154]. **b** Comparative liver parasite percent inhibition with m-DDSLNs in various doses and MFS (3 mg/kg) observed in *L. donovani* infected BALB/c mice model [154]. **c** Comparative in vitro percent inhibition of *L.* *donovani* amastigotes with paromomycin (PM), Amphotericin B (AmB), AmBisome®, AmB-SLNs and the surface modified drug loaded SLNs (m-DDSLNs) [154]. **d** Level of biochemical markers for renal toxicity (Urea & Creatinine) and for hepatotoxicity (ALAT & ASAT) followed by 14 days treatment with different modalities i.e., paromomycin (PM), Amphotericin B (AmB), AmB-SLNs and the surface modified drug loaded SLNs (m-DDSLNs) [154]

Polymeric nano-DDS is accompanied by numerous beneficial aspects in oral drug delivery. For instance, they are used to modify the pharmacokinetics and bioavailability of orally un-absorbable drugs. Furthermore, controlled rate and targeted drug delivery can be achieved by these nano-DDS [136, 145]. Thus, for effective oral delivery of conventional injectable anti-leishmanials, several polymeric nano-DDS have been designed. A recent study aimed to develop PEG-PLGA oral Amp-B NPs with prominent spleen, liver, and bone marrow parasite load reduction as compared to the injectable liposomal Amp-B. In this valuable experimental study, the pharmacokinetics of the designed oral nanosystem was compared with oral Fungizone®. AUC and C_max_ of PEG-PLGA NPs loaded with Amp-B was highly improved i.e., 12,325.1 μg.h/l and 480.2 ng/ml, as compared to Fungizone® having AUC and C_max_ of 9832.4 μg.h/l and 298.2 ng/ml, respectively. The oral bioavailability results of this study are, additionally, illustrated in Fig. 8. Improved oral bioavailability (GIT absorption) of Amp-B using PEG-PLGA nano-DDS is highly valuable because of speculated passive targeting of the hepatic leishmanial parasite. However, it should be evaluated, in vitro as well as in preclinical settings, to demonstrate its utilization against Leishmaniasis [146–148]. For PTM, trading-off between hydrophilic and lipophilic character is deemed to be necessary to compensate for its inadequate GIT absorption. This can be achieved through these, polymeric-based nano-DDS. These points are, however, supported by the research study carried out by Valle et al. [149] as they conducted multiple experiments to assess the release model and efficacy of PTM-PLGA-NPs. Orally administered NPs were found to exhibit a remarkable reduction in organ mass (liver and spleen) and parasitic burden in the murine model induced by *L. infantum* [44, 149]. In addition, multiple studies aiming to fabricate oral NPs have been conducted and some potentially important ones are mentioned in Table 3. These studies have, fortunately, proved the aptness of polymeric NPs as an effective nano-DDS with remarkable properties to intensify GIT absorption of anti-leishmanials, pictorially represented in Fig. 5b. Surfactant stabilized nanosized colloidal dispersion—nanosuspensions, can modify the dissolution properties of anti-leishmanials—ameliorate oral bioavailability by enhancing their water solubility [150]. Many studies have attempted to boost oral absorption and bioavailability of promising anti-leishmanial agent, Amp-B, by the utilization of nanosuspension. However, no such study has evaluated the Amp-B-nanosuspension activity against Leishmaniasis, neither in vitro nor in vivo [151, 152]. In 2016, Amp-B-SLNs has also been designed to promote its GI absorption and to escalate its oral bioavailability. Unfortunately, the results of the study were not much promising as AUC was 6063.11 μg.h/l which is lower than Amp-B loaded PEG-PLGA oral system as discussed earlier [148, 153]. Following the principle of cyclodextrin conjugation, surface modified SLNs have been fabricated facilitating the oral delivery of poorly absorbed anti-leishmanial agents—Amp-B and PMC. The SLNs (Amp-B-PMC-HPBC-SLNs) were modified using a 2-hydroxypropyl-β-cyclodextrin to enhance their intestinal permeability and absorption owing to its ability to impart hydrophilic character. Ample macrophage internalization has taken place, evaluated through confocal scanning microscopic technique. The cytotoxicity was also found to be 20% less than plain SLNs. Leishmanial IC_50_ has shown 10.07 times more efficacy than unmodified Amp-B-SLNs. These modified SLNs (abbreviated as n-DDSLNs in Fig. 9) were also presented with 95% *L. donovani* amastigotes internalized in J774A.1 macrophage, which was highest in comparison to Amp-B-SLNs, AmBisome®, and PMC. Moreover, kidney damage markers (creatinine and BUN) and hepatic damage markers (ALT and APT) were not elevated, demonstrating the safety of designed nanocarrier (Amp-B-PMC-HPBC-SLNs) post oral administration. This insightful study has also performed an in vivo anti-leishmanial assay against *L. donovani* in BALB/c mice model and compared the efficacy of oral Amp-B-PMC-HPBC-SLNs (20 mg/kg for 5 days) and compared with oral MFS (3 mg/kg for 5 days) and the liver parasitic percent inhibition was found to be 91.12% and 49.30%, respectively. Results of this study are represented pictorially in Fig. 9. Due to extensive characterization and anti-leishmanial activity comparison of this nano-DDS with currently employed treatment modalities (Amp-B, MFS, and PMC), this candidate could be selected for testing against a small human population [154]. This meaningful study has paved the way for modified nano-DDS and with more intense animal testing, this candidate can be enrolled in human clinical trials. An oral nano-DDS of PMC was designed, comprising of PLGA NPs modified with mannosylated thiolated chitosan, for imparting parasite selectivity [155]. Critical attention of researchers, however, is necessary to design more effective orally delivered anti-leishmanial nano-DDS towards achieving passive targeting and potential eradication of splenic and hepatic parasites.

**Table 3:** Potential Nano-DDS for oral delivery to curb Leishmaniasis

Type of Nano-DDS	Nanosystem engineered	Loaded anti-leishmanial drug	Characterization	Leishmanial strain	Animal model	Major results	References
Nano-dispersions	Nanosuspension	Amphotericin B	In-vitro	–	–	Enhanced Amp-B solubility and dissolution	[151]
Polymeric-based nanocarriers	PLGA NPs	Amphotericin B	In vitro + In vivo	*L. donovani*	BALB/c mice	Fabricated NPs comparable to that of injectable liposomal formulation in terms of the liver, spleen, and bone marrow parasitic burden reduction	[146, 147]
	Chitosan NPs	Rifampicin	In vitro	*–*	–	Biphasic drug release with 90% of drug release in 24 h at least	[156]
	PEGylated PLGA NPs	Amphotericin B	In vitro	*–*	–	Drug laden NPs have shown bi-phasic drug release (initial abrupt release followed by slow release)	[157]
	PLGA NPs	Pentamidine	In vitro + In vivo	*L. infantum*	BALB/c mice	0.4 mg/kg (totally 5 doses) of the fabricated NPs are associated with a valuable reduction in hepatomegaly and splenomegaly along with parasitic burden as well	[149]
Lipid-based Nanocarrier	HPBC Modified SLNs	Amphotericin-B + Paromomycin	In vitro + In vivo	*L. donovani*	BALB/c mice	Sustained release of the entrapped drug is observed with significant parasitic burden reduction in BALB/c mice model followed by oral administration Improved macrophage internalization, reduced toxicity, and enhanced anti-leishmanial efficacy	[154]

## Targeted delivery of anti-leishmanials—unfettered access to intramacrophage parasite

### Active targeting—NPs surface tuning approach

**Fig. 10:**
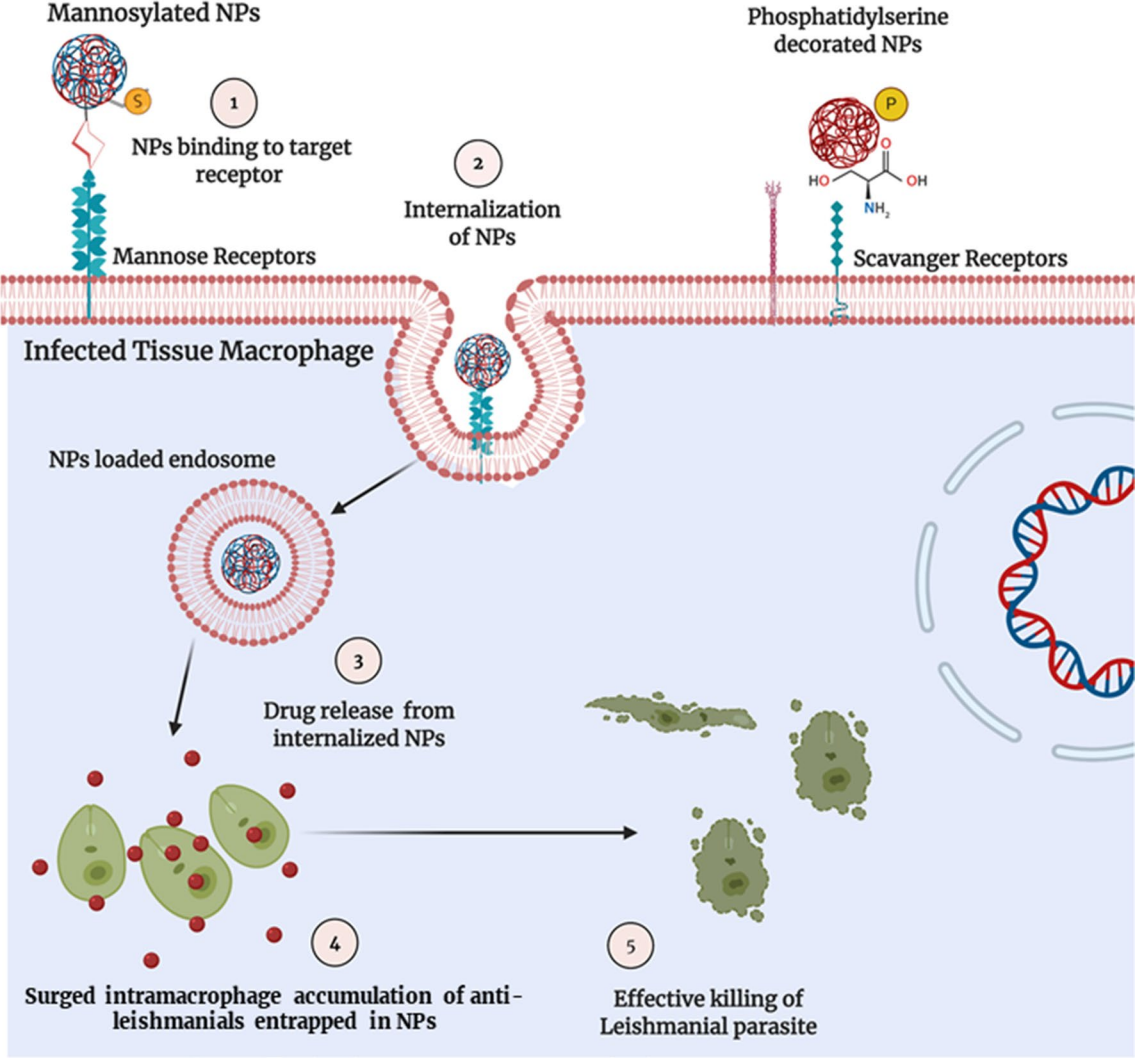
Surface-modified/antigen labelled NPs for enhanced drug accumulation and target killing of intra-macrophagial amastigotes; [1] Attachment of Mannose labeled anti-leishmanial nano-DDS with Mannose-receptors on macrophage membrane, [2] Receptor mediated endocytosis of Mannosylated anti-leishmanial nano-DDS, [3] Release of entrapped drug, [4, 5] Effective intramacrophage parasitic killing by Mannosylated nano-DDS

Active drug targeting is a valuable phenomenon opted for the purpose to achieve selective drug accumulation inside the organs, tissues, or cells of interest. Lower systemic toxicity has also been observed with actively targeted formulations. Also, the targeted formulations undergo a significant uptick in drug accumulation, as compared to untargeted moieties, inside desired targeted tissue [158, 159]. Nano-DDS with appropriate surface modification is an efficient system for active targeting of entrapped drug [160]. To concise our discussion towards Leishmaniasis, myriad surface labeling ligands have been used to target amastigote—a resident of tissue macrophage. These include d-Mannose, Phosphatidylserine, Lactoferrin, Chondroitin sulfate, and *p*-aminophenyl-α-d-Mannopyranoside. These ligand moieties are decorated at the NPs surface. This, in return, provide capability—the NPs are attracted towards their corresponding receptors (Mannose and Scavenger receptors). In detail, mannose-receptors (MR or CD206) are the glycoproteins, C-type lecithin, born by the membrane of tissue macrophages, RES macrophages, and dendritic cells. The outer (extracellular) portion of the mannose receptor comprises the cysteine-rich domain and carbohydrate recognition domain, which have a high affinity for sugars i.e. mannose, fructose, etc. [161]. Mannose attachment exteriorly to the NPs enhances its macrophage recognition and internalization. All these series of events, illustrated in Fig. 10, steer towards the intramacrophage accumulation of anti-leishmanial moiety. By now, several ligand-decorated NPs have been devised using the aforementioned surface modifying entities and shown to have compelling results in contrast to the sresults of previously engineered unlabeled nano-DDS.

#### Surface modified nano-DDS for active intramacrophage parasitic targeting

**Fig. 11:**
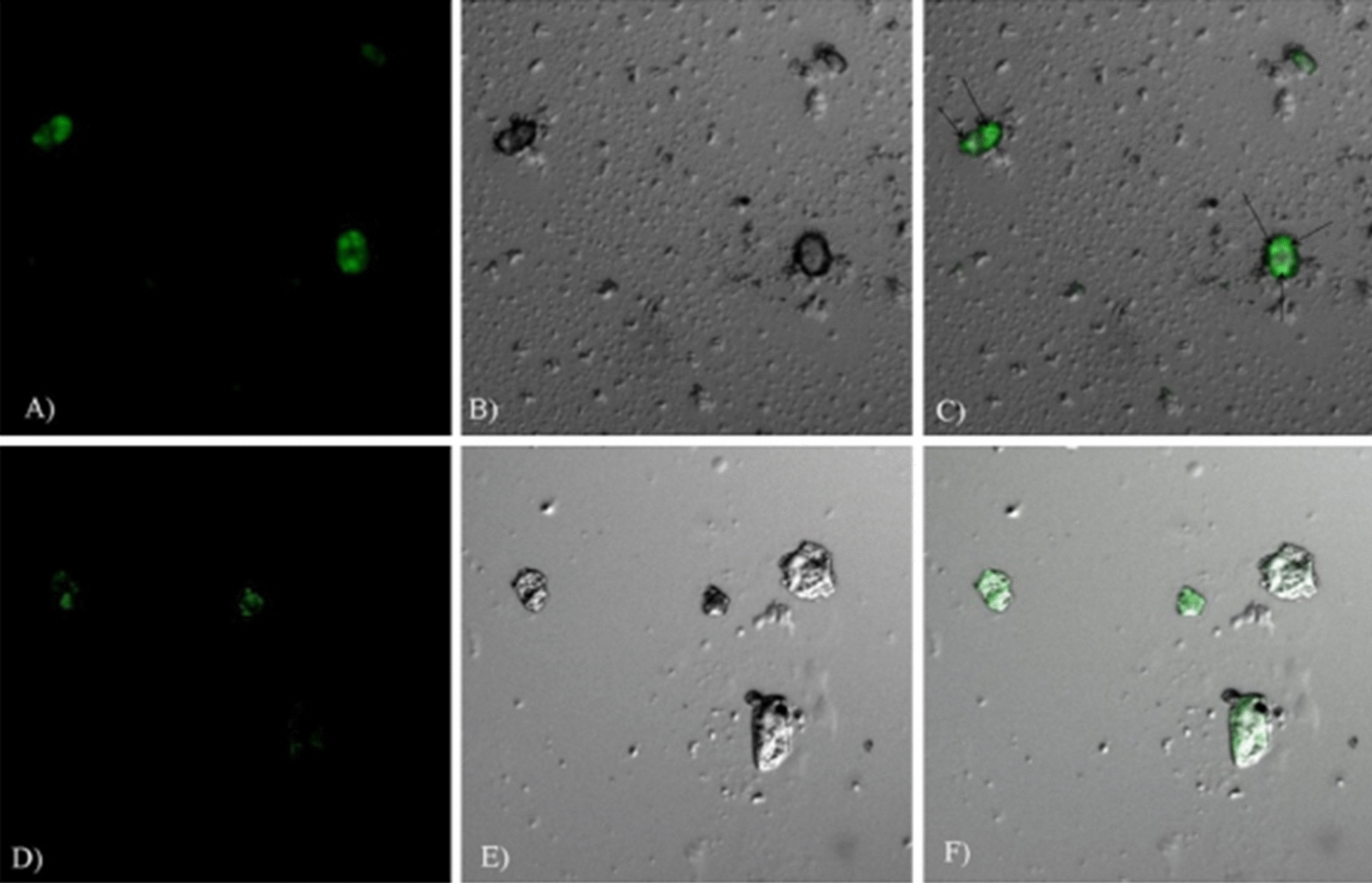
Confocal laser scanning microscope (CLSM) micrographs; **a**–**c** demonstrating significant macrophage internalization and accumulation of Fluorescein-mCNPs, **d**, **e** Indicating reduced macrophage internalization of CNPs [168]

The most usual approach opted for active targeting of anti-leishmanial agents is entrapping the conventional agent inside the lipid-based, polymeric-based, or other nano-DDS, followed by surface re-modeling through an attachment of d-Mannose—a ligand for Mannose receptor expressed by tissue macrophages [77]. In the previous decade, multiple attempts have been made to design surface tuned drug-loaded NPs as given in Table 4. Shahnaz et al. [162] designed Mannosylated thiolated chitosan (MTC) nanosystem loaded with Amp-B (Amp-B-MTC) having rod-shape, 362 nm particle size and 90% macrophage viability. Another worth mention finding was the retention of Amp-B-MTC inside reticuloendothelial system (RES) macrophage up to 10 days, confirming the effectiveness of thiolation. In addition, 1 μg/ml of AmBisome® and Amp-B-MTC respectively showed amastigote percent inhibition of 60% and 95%. The Amp-B-MTC fabrication was found to be a meaningful study showing IC_50_ values of 0.02 μg/ml as compared to Amp-B—0.26 μg/ml. In the *BALB/c* mice model, Amp-B-MTC was also found to be superior with 90% parasitic inhibition. Higher vesicular size of Amp-B-MTC was however of concern—as vesicles having a size from 100–300 nm [163] would experience optimum penetration macrophage internalization. These valuable results were highly suggestive for additional safety and efficacy assessment, to pursue its recruitment towards clinical trials. The inclusion of macrophage internalization assay would, however, effectively address this raised concern [162]. Moreover, the thiolation was speculated to reduce drug efflux via P-gp and TR-system inhibition. To improve macrophage internalization of Amp-B, another research study was carried out. They labeled CD-14 on the surface of formulated PLGA-PEG NPs [164]. Surface decorated PLGA NPs were also been engaged to enhance RES targeting of PMC by designing MTC-PLGA-PMC. In detail, the flow cytometric evaluation has shown the surged internalization of PMC in *L. donovani* infected J774A.1 macrophage. With MTC-PLGA-PMC, the concentration of PMC in 10^6^-macrophages was found to be 43.110 μg which was significantly higher than PLGA-PMC and plain drug i.e., 4.45 μg and 4.28 μg, respectively. The in vivo pharmacological assay was also supportive with a 3.6-fold increase in efficacy in terms of liver parasitic burden reduction. Additionally, in this study cytokine and NO evaluation was also carried out and displayed the highest IL-12 (600 pg) and INF-γ (550 pg) values in comparison to simple PLGA-PMC and plain PMC. Evaluation of NO level with a value of 85 μM has also exhibited the superiority of MTC-PLGA-PMC. Thus, the mannosylated system possesses more efficacy and anti-leishmanial response in contrast to the simple nano-formulation, as can be seen in case of the PLGA-PMC NPs [155]. As discussed earlier, the reduced efficacy of MA was due to the trypanosomatid TR-system mediated efflux. MA laden mannosylated thiolated polyethylenimine NPs have been investigated thoroughly and experimentally proved to have superiority over the plain drug owing to higher host RES uptake and inhibition of amastigote P-gp and other efflux pumps [165]. Lipid-based nano-DDS can also be tuned (surface modification) for active drug targeting and surged intra macrophage internalization [166]. Results of several recent studies stated erstwhile are elaborated in Table 4. However, the graphical results are visualized in Fig. 6. Additional ligands for APCs mannose receptors include lactoferrin, chondroitin sulfate, and *p*-Aminophenyl-α-d-mannopyranoside. Lactoferrin, an iron-binding glycoprotein, having a binding affinity with C-type lecithin receptors hold by macrophages and rest of APCs, due to the presence of *N*-glycosylation binding sites on lactoferrin. To enhance Amp-B RES intake, lactoferrin anchored PLGA NPs (LcPLGA-Amp-B) were designed. The designed formulation, interestingly, possessed promising results in terms of cure rates in *L. donovani* infected hamsters. LcPLGA-Amp-B have shown 2.15-fold intense internalization in *L. donovani* infected macrophages as compared to lactoferrin-free PLGA-Amp-B, evaluation was done through flow cytometry and confocal laser scanning microscopy. In vitro anti-parasitic study exhibited IC_50_ values of LcPLGA-Amp-B 3.20, 2.13, and 2.50 times higher than Fungizone®, AmBisome®, and PLGA-Amp-B, respectively. Fortunately, the levels of protective cytokines—IL-12, TNF-α, and INF-γ increased 2-times with LcPLGA-Amp-B. The safety parameter was assessed using acute toxicity demonstrated 12.5% mice killing as compared to Fungizone® (100%) and AmBisome® (37.5%) [167]. Designed rifampicin-loaded mannosylated chitosan NPs (R-mCNPs) also illustrated 2.31 and 16.2 times more macrophage internalization than the chitosan free NPs (CNPs) and conventional drug, respectively, and results of confocal scanning microscopy visualizing macrophage endocytosis are represented in Fig. 11 [168]. Another class of receptors responsible for the internalization of multiple entities; proteins and intruding pathogens, into APCs are the scavenger receptors (SR). A group of scientists has recently developed phosphatidylserine labeled MA-LPS (PS-MA-LPS). The anti-leishmanial assay was assessed against *L. major* amastigotes, promastigotes, and intracellular amastigotes, which displayed approximately tenfold enhanced anti-leishmanial response of the PS-MA-LPS displayed. Complete killing of intra-cellular amastigotes was experienced at 40-times lower concentration of PS-MA-LPS, in contrast to the free MA. Theoretically, the PS-MA-LPS trade showed enhanced intra-macrophagial accumulation due to SR mediated APC targeting [169]. Phosphatidylserine labeled doxorubicin-loaded nanocapsules (PS-DOX-NPs) exhibited 1.75-fold higher APCs internalization, significantly lowered IC_50_ (against *L. donovani*) along with elevated distribution in liver and spleen was also seen followed by administration in wister rats [77]. NPs apprehended with ligands of SRs were designed in attempt to promote intra-macrophagial drug accumulation which are mentioned in Table 4. Post critical evaluation of the above-mentioned research trials, it can be construed that surface labeled nanosystems are found to possess enhanced macrophage targeting and accumulation. This approach is, indeed, a value-added towards safety and efficacy as well. Anti-leishmanial nano-DDS, claiming the inhibition of TR-system and P-gp pumps, should also need to be assayed against animal models infected with MA/SSG resistant leishmanial strains. In this way, their preeminence can be evaluated properly in contrast to conventional anti-leishmanial nano-DDS. However, the designing of thiolated-mannosylated nano-DDS could be an expensive approach, mainly due to costly raw material and process complexity—for production of mannosylated thiolated polymer [155].

**Table 4:** Surface modified Nano-DDS for target leishmanial destruction

Nanosystem engineered	Decorated ligand	Target receptor protein	Loaded anti-leishmanial drug	Characterization (Animal model)	Major results	References
Chitosan NPs	d-Mannose	Mannose receptor expressed by macrophages	Amphotericin-B	In vitro (*L. donovani* infected J744 macrophages) In vivo (*BALB/c* mice)	Mannosylation has resulted in persistent intramacrophage drug accumulation up to 10 days The designed mannosylated thiolated system exhibited better internalization and intramacrophage amastigote killing activity as compared to L-Amp-B	[162]
	d-Mannose	Mannose receptor expressed by macrophages	Rifampicin	In vitro	Macrophagial internalization of drug-loaded in mannose labeled NPs was observed about 2.31-fold higher than unlabeled chitosan NPs	[168]
	d-Mannose	Mannose receptor expressed by macrophages	Paromomycin	In vitro (*L. major* amastigote infected macrophages)	The strong leishmanicidal response was observed with mannosylated chitosan nanocarrier, owing to its targeting ability-lowest IC_50_ No toxicity was observed against THP-1 cells-highest CC_50_	[170]
Thiolated Polyethylenimine NPs	d-Mannose	Mannose receptor expressed by macrophages	Meglumine Antimonate	In vitro (*L. tropica* infected macrophage)	Mannosylation has resulted in enhanced accumulation of drug inside infected macrophages Thiolation has resulted in inhibition of P-gp and TR-system	[165]
Nano lipid carriers (NLCs)	d-Mannose	Mannose receptor expressed by macrophages	Rifampicin	In vitro (2447-*Mycobacterium avium* infected macrophages derived from C57B1/6 B6 mice)	Mannosylated NLCs are more beneficial in terms of bacterial killing as compared to non-mannosylated NLCs and plain drug. Mannosylated NLCs exhibit lower IC_50_	[166]
Thiolated Chitosan-PLGA NPs	d-Mannose	Mannose receptor expressed by macrophages	Paromomycin	In vitro (*L. donovani* infected macrophages) In vivo (*BALB/c* mice infected with *L. donovani*)	Simultaneous mannosylation and thiolation was associated with boosted cellular uptake of drug and lower incidence of efflux Nitric oxide production evaluation also yields the results evidencing its supremacy to non-thiolated and non-mannosylated NPs	[155]
Gelatin NPs	d-Mannose	Mannose receptor expressed by macrophages	Amphotericin-B	In vitro (J74A.1 macrophage cell line infected with *L. donovani* amastigotes)	Mannosylated gelatin NPs appeared to hold enhanced intramacrophage parasitic inhibition to that of AmBisome®	[171]
PLGA-PEG NPs	CD-14	–	Amphotericin-B	In vitro (*L. donovani*) In vivo (Hamsters)	Better splenic parasitic reduction with Amp-B loaded PLGA-PEG CD-14 bio-conjugated NPs as compared to the conventional Amp-B and Liposomal Amp-B	[164]
PLGA NPs	Lactoferrin	Mannose receptor expressed by APCs	Amphotericin-B	In vitro (J744A.1 macrophage cell line infected with *L. donovani*) In vivo (Hamsters)	Lactoferrin labeled Amp-B loaded nanocarrier was resulted to have lowest IC_50_ and the highest percentage of amastigotes execution than rest of the experimental formulations	[167]
Liposomes	Phosphatidylserine	Scavenger receptors (SR) expressed on the surface of macrophages and other APCs	Antimony (Sb)	In vitro (M 6445—*L. chagasi*	IC_50_ of liposomal Sb is 16 times lower than that of conventional pentavalent antimony However, the results of leishmanial infected macrophage drug internalization were not much satisfactory	[172]
Nano-capsules (NCs)	Phosphatidylserine	Scavenger receptors (SR)	Doxorubicin	In vitro (J744A.1 macrophage cell line infected with *L. donovani*) In vivo (Wister Rats)	Results were satisfactory with 1.75-fold higher APCs internalization, significantly lowered IC_50,_ and higher distribution in liver and spleen is seen followed by the administration in Wister rats	[173]

### Anti-leishmanial nano-DDS—a passive targeting strategy as well

Contrary to active targeting, passive targeting is just a spontaneous targeting of cells, tissue, or organ, based upon the enhanced permeation of designed NPs. This enhanced permeation would result in enough tissue accumulation sufficient to eradicate the underlying pathological conditions. It is usually attained through alteration in physicochemical and pharmacokinetic properties of the drug [174]. An advanced way to achieve passive targeting is the use of nanomedicine—nanocarrier-based drug delivery system. In Leishmaniasis, a passive targeting technique has been employed for both CL and VL forms of the infection. The orally designed polymeric NPs undergo intestinal absorption through M-cells in the peyer’s patch that enables them to internalize in epithelial APCs. Moreover, adequate intestinal absorption of drug-loaded nanosystem would result in appreciable internalization and accumulation of anti-leishmanials in damaged liver and spleen [32]. The orally administered nano-DDS are previously discussed in detail in “Promising nano-DDS—capable for oral delivery of injectable anti-leishmanials” section and Fig. 5b. WHO has recommended the use of topical dosage forms for the treatment of uncomplicated CL. Topical application is beneficial because of better acceptability and less systemic toxicity. However, several limitations still encounter with cutaneous/topical drug delivery. A major challenging factor for topical drug delivery is inadequate permeation of drug through densely packed skin barrier—stratum corneum (SC) [175]. This could be the possible reason for inadequate parasitic eradication in CL patients.

#### Passively target nano-DDS for CL

**Fig. 12:**
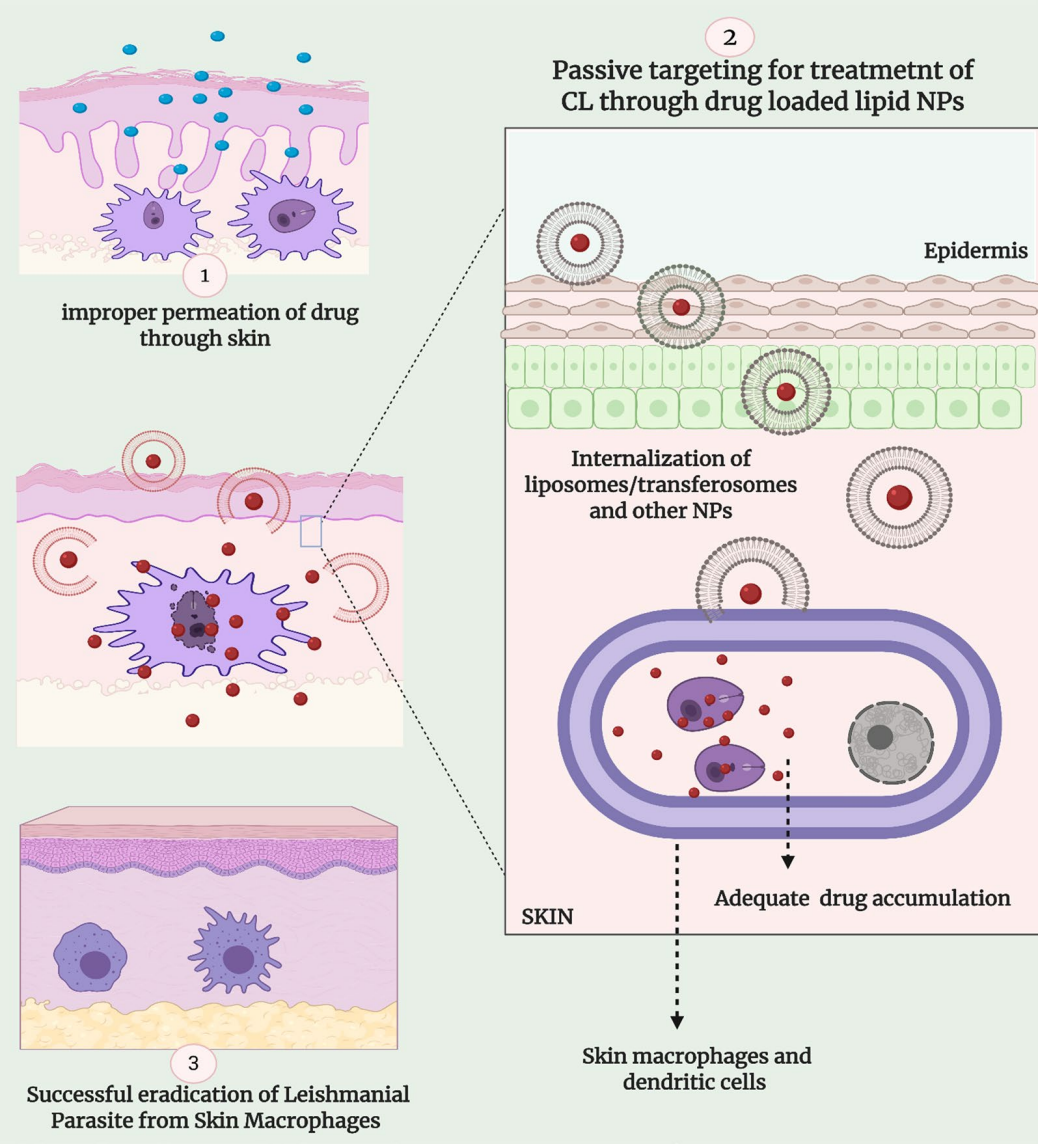
Aggravated Skin permeation, intra-macrophagial internalization and target execution of leishmanial parasite responsible for CL by topically applied nano-DDS

In the former decade, the evolution of topically applied nano-DDS has also resolved the permeation challenges. Nano-sized, deformable lipid-based vesicular NPs are the key candidate for topical delivery [176]. Moreover, the topically administered drug target passively the CL parasite, illustrated in Fig. 12, as the absorbed drug internalize and accumulate selectively inside the skin APCs infected with the leishmanial parasite. Lipid-based NPs, particularly the TFS, experience the least hindrance while crossing epithelial skin to reach underneath diseased macrophages [177]. In addition, other types of nano-DDS can also have the potential of permeating through deeper layers of the epidermis. Niosomes, nano-emulgels, and microemulsion have also been evaluated as a topical nanosystem for passive CL targeting [178–180]. Topical nano-DDS are advantageous as a consequence of lower systemic permeation of anti-leishmanials. This would ultimately reduce the incidence of toxicity as well as parasitic resistance. In 2014, the first study was reported which has evaluated the response of topical LPS formulation of MA (MA-LPS). Only 1.5% of MA-LPS permeate while 65% retained in the skin, assessed through in vitro skin permeation test. ED_50_ against amastigotes (*L. major*) was found to be 46.36 μg/ml, significantly higher than the plain drug. The efficacy against *L.* major induced *BALB/c* leishmanial model was however surprising. Till 4 weeks of treatment (50 mg twice daily), the lesion size gets reduced. The treatment discontinuation, unfortunately, rehabilitates the lesion size. This could be due to resistance development in *L. major* amastigotes [181]. In another published report, Amp-B-LPS were designed and shown to have skin penetration only of 4% supplemented with 73.92% retention. Followed by the treatment with 0.4% Amp-B-LPS complete skin and splenic parasitic eradication was achieved [182]. In a similar study, MFS-LPS exhibited only 3% permeation through the skin. Accompanied with dodging the needless drug permeation, lesion size and splenic and skin lesion parasitic minimization were highly significant (p < 0.001), contrary to plain LPS and drug [183].

Owing to the inadequate treatment response, topical LPS were modified into TFS gels, designed extensively in the last 5 years. These nano-deformable systems have better potential to cross SC owing to their elastic nature. Dar et al. [163] designed SSG-TFS and found to have a deformability index of 43%. Macrophage cytotoxic test demonstrated the safety of the designed nanosystem and displayed CC_50_ values of SSG and SSG-TFS as 1.65 and 1.3 mg/ml, respectively. IC_50_ value of SSG-TFS was found to be 50.86 μg/ml in comparison to the plain SSG (184.66 μg/ml) representing marked efficacy. Moreover, to support this in vivo characterization was also conducted which exhibited significant lesion size reduction (1.92 ± 0.31 mm) as compared to the plain SSG treatment group (5.03 ± 0.44 mm) [163]. In another recent report, the rifampicin-TFS gel was designed, having deformability of 93%, and evaluated for its usefulness against CL. In addition to its sustained release behavior, the intramacrophage buildup was also significantly enhanced as compared to the plain drug, Moreover, flow cytometry assay resulted in 0.3% viable cells only with rifampicin-TFS [177]. Their deformable nature departs anti-leishmanial agents more impressively to intramacrophage cutaneous parasites, as proven by the previously mentioned results. Hence, these elastic nano-DDS have the potential to improve treatment outcomes of CL and other cutaneous anomalies. Further estimation of safety and efficacy would determine the fate of these promising nano-DDS. For comparative analysis, their in vivo anti-leishmanial activity should be compared with PMC, as a reference standard, because it is the only available drug for topical application in CL patients [40]. Furthermore, the assessment of lesion parasite reduction is not sufficient as Leishmania can be disseminated to the spleen and liver, causing hepato-splenomegaly [184]. Additional details of recently fabricated lipid nano-formulations (SLNs, LPS, and TFS) are discussed systematically in Table 5 along with their results and outcomes.

**Table 5:** Lipid-based drug-loaded Nano-DDS of Passive CL targeting

Nanosystem engineered	Loaded anti-leishmanial drug	Core components for fabrication	Characterization	Animal model	Major results	References
Liposomes	Meglumine Antimonate	Sphingosylphosphorylcoline Cholesterol Propylene glycol	In vitro + In vivo	*L. major* infected *BALB/c* mice	Designed nano-formulation was associated with significantly lower lesion size and splenic parasites post-treatment	[181]
	Amphotericin-B	Phosphatidylcholine Cholesterol	In vitro + In vivo	*L. tropica* infected *BALB/c* mice	Topical L-Amp-B 0.4% was found with the capability to complete eradication of skin lesions and splenic parasites as well	[182]
	Miltefosine	Phosphatidylcholine Cholesterol Propylene glycol	In vitro + In vivo	*L. major* infected *BALB/c* mice	Lesion size and parasitic burden reduction with 2% and 4% MFS loaded LPS	[183]
Transfersomes	Sodium Stibogluconate	Phospholipon G Tween 80	In vitro + In vivo	*L. tropica* infected *BALB/c* mice	Drug loaded TFS shown to have tenfold improved deep epidermal permeation and fourfold lower IC_50_ as compared to the plain drug solution	[163]
	Rifampicin	Phospholipon G Span 60 & 80 Tween 20 & 80 Sodium lauryl sulfate	In vitro + In vivo	*L. tropica* infected *BALB/c* mice	Lower IC_50_ (for leishmanial cellular apoptosis) of rifampicin loaded TFS was observed	[177]
Nano-structured lipid Carriers	Amphotericin-B	Phospholipon G Isopropyl myristate Glyceryl monosterate	In vitro + In vivo	*L. major* infected *BALB/c* mice	Significant parasitic burden reduction in leishmanial lesion	[185]

## Nanosystem based modalities to curb parasitic resistance

Numerous nanosystem based schemes have been employed to tackle leishmanial parasitic resistance against the fundamental treatment approach—the chemotherapy. Active drug targeting, already discussed in detail, has been evaluated as a reasonable approach exhibiting appropriate intra macrophage accumulation of various leishmanicidals. In return, this could prevent the resistance and survival of the parasite. Thiolation is another excellent means to minimize the risk of resistance emergence discussed later. Another practical approach to head towards the formerly stated aim is the entrapment of multiple (more than one) anti-leishmanial drug inside the nanosystem. The delivery of multiple drugs loaded NPs (co-loaded NPs) not only improves drug efficacy but also prevents the rapid expulsion of drugs because of simultaneous leishmanial apoptosis in numerous ways. The details of these strategies are discussed below along with pictorial representation in Fig. 4b.

### Thiolated Nano-DDS—inhibitors of trypanothione (TR) metabolism

**Fig. 13:**
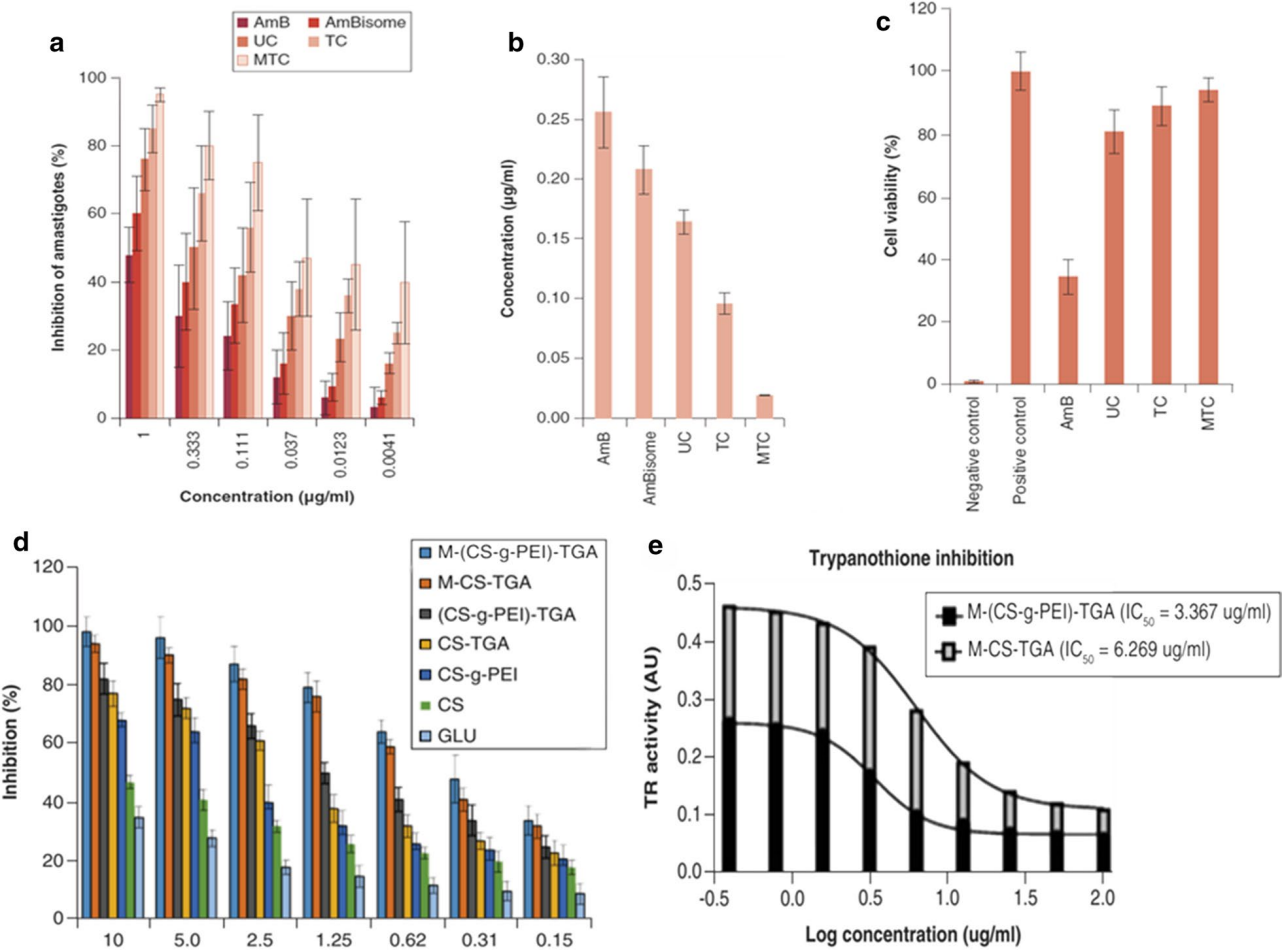
**a** Percent inhibition of amastigotes of Amp-B-MTC at various concentrations and comparison with plain thiolated nanosystem (TC), non-thiolated nanosystem (UC), AmBisome and Amphotericin B (AmB) [162]. **b** IC_50_ of Amp-B-MTC against L. *donovani* amastigotes [162]. **c** Comparative cell viability assay showing safety of Amp-B-MTC [160]. **d** In vitro anti-leishmanial response in terms of—Inhibition (%) of mannosylated thiolated polyethylenimine nano-DDS. against L. *tropica* amastigotes at various concentrations [163]. **e** Trypanothione reductase inhibition at multiple log concentrations of designed polymers with their respective IC_50_ [165]

The practical mechanism for parasite protection against numerous toxins, drugs, and oxidative stress—the TR-system, when suppressed would result in adequate drug internalization and enhancement of nanosystem efficacy. Thiomers—the polymers, have properties to impart sustained release behavior in consort with enzymatic and efflux pump (P-gp) inhibition abilities. Nano-DDS utilizing these polymers are commonly termed as “thiolated” nano-DDS. To study the effect of thiolated nano-DDS for curtailment of the resistant leishmanial strains, MA loaded mannosylated thiolated polyethylenimine chitosan NPs (MA-MTPC-NPs) were designed and evaluated against MA resistant leishmanial strains. Sarwar et al. [165] reported that MA-MTPC-NPs were indicated with the highest meglumine internalization in uninfected macrophages, resistant-strain infected macrophages, and sensitive-strain infected macrophages, 61.47, 61.61, and 61.76 μg MA/10^6^ macrophages, respectively, in comparison to the plain drug and non-thiolated NPs. Published results of various thiolated nano-DDS are illustrated in Fig. 13. The superiority of other designed anti-leishmanial thiolated nanosystem is discussed erstwhile in section “Surface modified nano-DDS for active intramacrophage parasitic targeting” and mentioned systemically in Table 4, in the “Major results” column. The expulsion intensity of several ABC transporters in the leishmanial membrane can also be reduced by employing thiolated nano-DDS [162]. Several studies have been carried out to evaluate the intra macrophage residence of thiolated nano-formulations, mentioned in “Anti-leishmanial nano-DDS—a passive targeting strategy as well” section. Amp-B and PMC thiolated nano-formulation have been designed and evaluated. Results have shown 71 and 12.73-fold higher macrophage internalization of the fabricated nano-formulations as compared to the free drugs solutions, respectively [155, 162]. Silver (Ag) NPs also retained activity for suppression of parasite protective mechanism—the TR-system. A group of Italian scientists has investigated the inhibition potential of silver on the trypanothione reductase activity of the leishmanial parasite. As an indicative of the leishmanicidal effect of AgNPs, IC_50_ for amastigotes and promastigotes were 1.76 and 2.18 μM, respectively. Hence, AgNPs exhibited strong leishmanial-toxic effects [77, 186].

### Co-loaded nano-DDS for Leishmaniasis

**Fig. 14:**
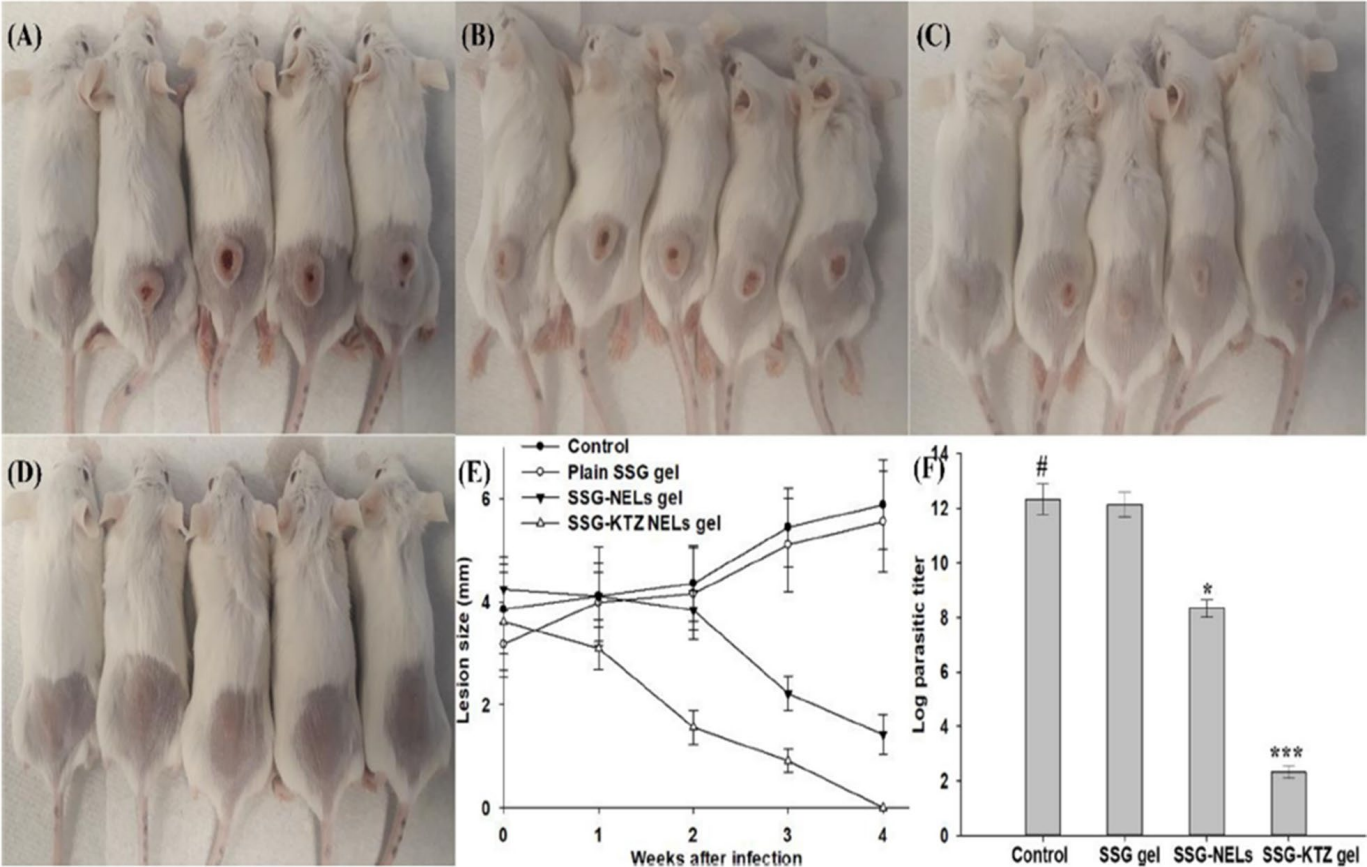
Back rump CL lesion of various groups of infected BALB/c mice model **a** Control, **b** Plain SSG gel, **c** SSG-TFS gel, **d** SSG-ketoconazole-TFS gel. **e** CL lesion size evaluation post treatment with various formulation with highest response with SSG-ketoconazole-TFS gel (SSG-KTZ gel). **f** Significant lesion parasite minimization with SSG-ketoconazole-TFS gel (SSG-KTZ gel) [190]

**Fig. 15:**
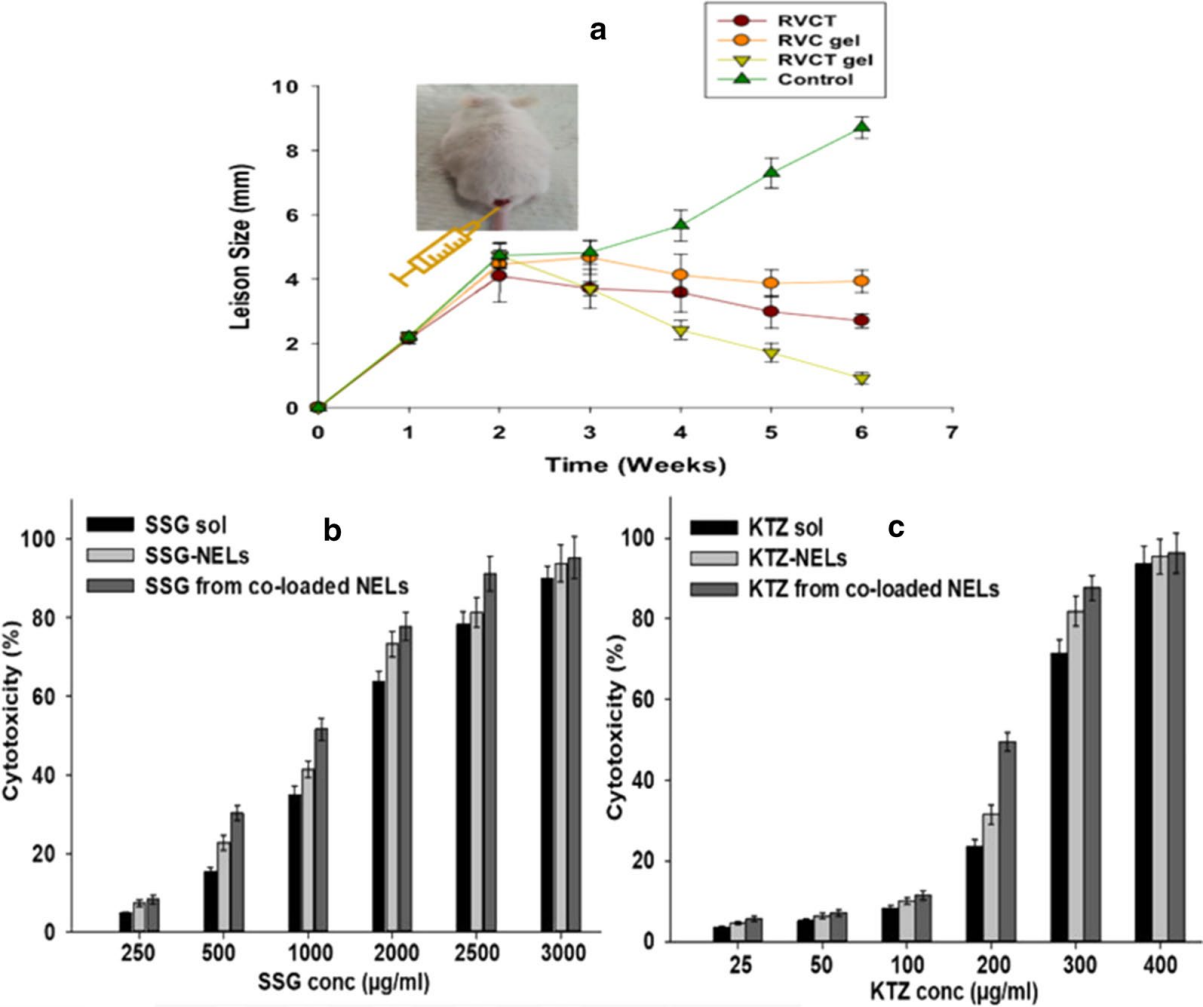
**a** CL lesion size reduction in infected BALB/c mice model followed by treatment with RV-TFS (RVCT) [192]. **b** Cytotoxicity comparison of SSG solution (SSG sol), SSG-TFS (SSG-NELs) and SSG-Ketoconazole TFS (SSG-KTZ-NELs) [190]. **c** Cytotoxicity comparison of KTZ solution (KTZ sol), KTZ -TFS (KTZ -NELs) and SSG-Ketoconazole TFS (SSG-KTZ-NELs) [190]

Anti-leishmanial monotherapy is considered one of the risk factors for the occurrence of parasitic resistance. Thus, combination therapy with multiple agents is needed not only due to increased anti-parasitic response, but also to prevent the emergence of resistant leishmanial strains in several regions of the world particularly in India and Brazil [187]. Natural immunomodulators, Curcumin (CUR), enclosed in PLGA-NPs had shown to increase MFS in vitro as a well in vivo anti-leishmanial activity when used together orally for VL. Approximately, 90% parasitic count was reduced with combination use of CUR-PLGA-NPs and MFS on 28th-day post-treatment along with significant elevation of ROS and reactive nitrogen species [188]. MA loaded chitosan-TiO_2_ NPs (MA-CTiO_2_-NPs) have been designed for synergistic killing of *L. major* promastigotes and intra-macrophage amastigotes. These nano-assemblies were found to be superior over alone MA treatment with respect to enhanced apoptotic potential, 4- and 13-fold, against amastigote and promastigote, respectively [189]. To sum up, the treatment with multiple agents could be beneficial in resistant VL cases. To face-off, the emergence of CL resistance, duo drug-loaded lipid-based nano-DDS were designed in past few years. These co-loaded topically applied nano-formulations were not only benefited with resistance prevention but also associated with victorious tissue parasite eradication at significantly lower IC_50_ in *L. mexicana* infected *BALB/c* mice model. Dar et al. [190] reported MFS-ketoconazole co-loaded-TFS (MK-TFS) with excessive *L. mexicana* killing assessed through flow cytometry assay. Moreover, in comparison to free SSG, in vitro anti-parasitic experiments have shown 10.67-fold reduced IC_50_ of MK-TFS as well as 35.33-times more parasitic-density reduction in the skin lesion. In vivo and in vitro results of this study are shown in Fig. 14 [190]. Similarly, MFS-Amp-B-TFS were designed by the researchers to assess the synergistic response against CL. Furthermore, these nano-DDS were evaluated for parasitic reduction through in vitro and in vivo experimentations. The IC_50_ value of designed MFS-Amp-B-TFS showed 8.62 and 5.22 times reduction contrary to Amp-B and MFS, respectively [191]. Recently, vancomycin and rifampicin were entrapped inside these deformable nanocarriers (RV-TFS) and evaluated for their leishmanial inhibition response. In addition to the sustained release behavior of the enclosed drugs, IC_50_ values of RV-TFS were found to be 1.41 and 1.52 μg/ml against *L. tropica* promastigotes and amastigotes, respectively. These values were significantly higher than the values of the individual drugs. Hence, due to better release profile and synergistic effect RV-TFS, better provision of parasitic curtailment occurred as presented in Fig. 15a [192]. This strategy could be beneficial as small doses of individual drugs are provided, hence, better results could be achieved with minimal chances of toxicity. The details of these studies, investigations, and explorations are mentioned in Table 6.

**Table 6:** Promising Nanosystem candidates, with eminent in vivo results, to curb leishmanial resistance

Nanosystem engineered	Loaded anti-leishmanials drug	Year	Route of administration	Leishmanial strain	Animal model	Major results	References
Stearylamine bearing Liposomes	Amphotericin-B	2008	Parenteral	*L. donovani*	VL induction in *BALB/c* mice	SA bearing Amp-B LPS are superior to AmBisome in parasitic burden reduction, IL-10, and TNF-α downregulation	[193]
	Paromomycin	2011	Parenteral	*L. donovani*	VL induction in *BALB/c* mice	The prominent synergistic response was observed evidenced by elevation of INF-α levels and reduction in IL-10 levels	[194]
	Sodium Stibogluconate	2011	Parenteral	SSG-resistant strains of *L. donovani*	VL induction in *BALB/c* mice	Successful destruction of SSG-resistant strain of *L. donovani* with the significant rise in NO levels	[195]
Transferosomes	Miltefosine + Apigenin	2019	Topical	*L. Mexicana*	CL induction in *BALB/c* mice	With co-loaded nano-formulation, 8.0-fold lower IC_50_ and 9.5-fold eradication of lesional parasite than plain MFS solution	[196]
	Amphotericin-B + Miltefosine	2020	Topical	*L. Mexicana*	CL induction in *BALB/c* mice	Significant reduction (8.6 & 5.22-times) in IC_50_ as compared to the plain drug solution of Amp-B and MFS respectively	[191]
	Sodium Stibogluconate + Ketoconazole	2020	Topical	*L. Mexicana*	CL induction in *BALB/c* mice	10.67 times lower IC_50_ was observed with fabricated nano-formulation and 35.33-times lesional parasitic load reduction was observed in comparison to plain SSG	[190]

## Distinctive nano-DDS for Leishmaniasis

### Photosensitive and inorganic nano-DDS

Owing to their capability to generate reactive oxygen moieties (ROS), metallic NPs possess clinically significant leishmanial agents killing activities. Although the presence of an anti-oxidative TR-system, that tries to cope with the ROS species, still parasitic damage encounters. Two studies have shown that AgNPs and TiO_2_-NPs are suitable alternative candidates for Leishmania treatment. The former study reported a 1.5 to 3 times reduction in parasite metabolic and proliferating ability in the dark and 2–6.5 times lowering in presence of UV-light [197]. Moreover, the parasitic external and internal morphology was also damaged, followed by the treatment with AgNPs. In fact, it possessed the strongest leishmanicidal activity among all metallic NPs. Cu-doped ZnO-NPs are also shown with surge ROS level, intra-parasitically, after exposure to visible light [198]. On the similar proof-of-concept, Zn-doped-Ti0_2_-hypericin photosensitive nano-DDS formulated by Sepúlveda et al. [199] demonstrated that the mixture of red and blue light posing an intensity of 22 mW/cm^2^ caused 43–58% reduction in lesion parasitic burden in *L.* *amazonensis* infected *BALB/c* mice CL model. Moreover, its effectiveness as an antileishmanial nano-DDS candidate was found to be comparable with Amp-B. Photodynamic nano-DDS can be a suitable alternative to conventional anti-leishmanials. However, more advanced in vitro and preclinical assessment is required to promote their utilization in Leishmaniasis [199]. Another innovative approach, Amp-B-Ag NPs, were designed and investigated for in vitro anti-leishmanial activity. The results were declared satisfactory with significant potency and uptick in ROS. Moreover, the activity was significantly enhanced following exposure to visible light [200]. The combined application of *Nigella sativa* oil and TiO_2_-Ag-NPs showed enhanced anti-leishmanial response against both forms of the parasite [201]. A drug with anti-viral, anti-inflammatory and anti-tumor potential, betulinic acid, has been entrapped inside chitosan NPs for evaluation regarding its safety and efficacy in *L. major* (resistant strain) infected *BALB/c* mice. The results of the study were found to be very satisfactory in terms of parasitic burden reduction and nitric oxide (NO) production. Approximately, 86% killing of promastigotes, 81% lowering in the intracellular parasite, and highest NO production was observed with this fabricated nanosystem [202]. Recently, manganese oxide NPs have been formulated for the purpose to search for alternative treatment modalities against Leishmaniasis. This metallic nano-DDS was associated with 57% promastigotes apoptosis, estimated using flow cytometry, at IC_50_ of 15 μg/ml. Metallic nano-DDS was believed to be cytotoxic in nature, thus, to assess its safety MTT assay was conducted and it was found to be toxic to macrophages at 40 μg/ml [203]. Metallic NPs are toxic at higher doses, thus, the use of anti-leishmanial loaded metallic nano-DDS could be effective even at non-toxic lower concentrations.

### Phytonanotechnology against leishmaniasis—a bio-friendly and cost-effective approach

Synthetically fabricated NPs are accompanied by multiple drawbacks as discussed in “Loopholes in designed nano-DDS” section. Nevertheless, these “nature’s nano-DDS” have enumerated myriad dilemmas associated with “synthetic nano-DDS”. This statement is supported by the experimental data stating “plant-derived nano-DDS” are eco-friendly, bio-friendly (non-toxic), and cost-effective [204]. Botanically-obtained isolates are currently being used for no-toxic and eco-friendly synthesis of various metallic NPs. This newly emerging arena is referred to as “Phyto-nanotechnology” [205]. The use of phyto-nano-DDS to curtail anti-leishmanial chemotherapy challenges is another appropriate way out. Saponins are the triterpene glycosides having intrinsic anti-biological, anti-infective, anti-leishmanial, and anti-inflammatory activity [206, 207]. An attempt was made to engineer Aescin-entrapped PLGA NPs. After accomplishment of the study, the results were clearly pointing towards improvement of intra-macrophage trafficking of designed phyto-nanosystem along with twice reduction in IC_50_ value as compared to blank aescin [208]. Anti-biological activities of propolis, a biotechnological product attained from bees (*Apis mellifera*), advocated the group of Brazilian and English scientists to design propolis loaded nanosystem. Brazilian red propolis extract was encapsulated inside the polymeric NPs constructed using poly-caprolactone and pluronic. This polymeric formulation had particle size in 200–300 nm range, inhibited *L. braziliensis* parasite at significantly lower IC_50_ values along with improved anti-oxidant properties contrary to the ethanolic extract [209]. Experiments conducted by Monzote et al. [210] revealed the anti-leishmanial activity of essential oil derived from *Artemisia absinthium*. Later, nanochelates (74 nm in diameter) of *A. absinthium* extracted essential oil was designed to gauge their anti-leishmanial response via duo in vitro and in vitro experiments. Results of both studies were conflicting; adequate lesion size reduction was achieved, but in vitro anti-leishmanial activity was limited with higher IC_50_ values [211]. By critical analysis of the results, the designed nanochelates were found appropriate channel for safer delivery of the plant extracts. Ocotea duckei vattimo, a Brazilian plant extract laden SLNs have also been extensively evaluated and found to be significantly parasiticidal to Leishmania [212]. CUR, a polyphenol enriched inside *curcuma longa* rhizomes had also displayed anti-leishmanial activity, as reported by a group of researchers from Pakistan [213]. They loaded CUR in NLCs for topical application against CL. In vitro anti-promastigote experiments yielded reduced IC_50_ readings of CUR-NLC gel as compared to the plain CUR-solution i.e. 105 μg/ml and 165 μg/ml, respectively [214]. Similarly, due to leishmanicidal activity of *Cupressus semipervirens* isolates containing cedrol, its NLCs have been engineered. Myriad in vitro and in vivo characterization have shown the superiority of cedrol-complexed-NLCs over plain cedrol extract and MTF. Moreover, its anti-leishmanial activity was also evaluated against SSG and PMC AG83-R and GE-1 amastigotes, in addition to the in vivo assay [215]. Another triterpene—ursolic acid, has been entrapped chitosan shielded NLCs, because of its ability to exhibit apoptotic changes in the leishmanial parasite [216]. Evaluation of leishmanicidal response was assessed by both in vitro and in vivo assays. In contrast to the free ursolic acid, the anti-leishmanial activity was increased by 12-fold against wild stains and fourfold against SSG and PMR resistant strains. Moreover, these in vitro results are supported by outcomes of in vivo assay—approximately 99% reduction in parasitic burden post oral administration of ursolic acid-loaded NLCs [217]. The instability of lipid nano-formulations was tackled using Aloe Vera leaf extract nano-assemblies. This novel methodology was presented with reduced nephrotoxicity and hemolysis as well as the nanosystem stability also improved to a greater extent. Its therapeutic efficacy was evaluated against fungal infection (caused by *candida *spp.), however, its anti-leishmanial response needs to be evaluated [218]. In literature, the extensive parasiticidal activity of metallic NPs has been reported. Unfortunately, they were synthesized usually either by physical or chemical method. These methods have incorporated multiple drawbacks in the use of metallic nano-DDS [219]. Phytonanotechnology, as described earlier, has resolved multiple drawbacks, through the employment of biogenesis of nano-DDS. In the current decade, silver and gold nanoparticles have been synthesized through an eco-friendly method—green synthesis and evaluated for their anti-leishmanial response [205]. *Isatis tinctoria* extract was loaded inside AgNPs and the IC_50_ value was determined against *L. tropica* promastigotes. Solo use of AgNPs presents IC_50_ values of 4.2 μg/ml and with the combined use of Amp-B, the IC_50_ was reduced to 2.43 μg/ml [200, 220]. In another recent report, AgNPs were designed with *Euphorbia prostrata* leaves extract. The in vitro leishmanicidal assay generates the IC_50_ values of 3.89 and 14.94 μg/ml against *L. donovani* amastigotes and promastigotes, respectively [221]. Similarly, Kalangi et al. (2016) [222] have found that AgNPs encapsulated *Anethum greveolens* leaves extract (35 nm in size) was less effective, when used alone. However, its anti-leishmanial response increased twice when employed in MFS doped form (combination use). Ag-Au hybrid NPs were also designed using Dioscorea bulbifera with a particle size of 15 nm. This hybrid approach was found to exhibit a significant anti-leishmanial response i.e. MIC was 32 μg/ml [223]. Extract of *Maytenus royleanus* and *Sargentodoxa cuneata* was also exercised to design AuNPs. Promastigote growth inhibition of 75% and IC_50_ of 5.29 μg/ml were observed, respectively [224]. In a recently published report, Bagirova et al. [225] have synthesized and compared the leishmanicidal response of AgNPs with green synthesized AgNPs using *Cuminum cyminum* seed extract. The biosynthesized AgNPs were superior for safety (with 12% of cell viability) as well as efficacy against *L. tropica* in comparison to plain AgNPs (with 2% cell viability). Unfortunately, in vivo leishmanicidal assay was missed in this published research report. To shorten the discussion, the nano-DDS utilizing substances of natural origin are quite beneficial as they provide an opportunity to recuperate the Leishmaniasis management. However, there alone use would not be recommended because of their limited anti-leishmanial activity. Designing co-loaded nanosystem enclosing plant extract and anti-leishmanials in small doses would be appropriate for successful killing of parasite.

### Innovative nano-DDS for Leishmaniasis

Tocopheryl polyethylene glycol 1000 succinate, also termed TPGS, was a P-gp inhibitor and prevent the drug efflux from parasite [226, 227]. In 2018, a novel nano-DDS consisting of Oleanolic acid-loaded PLGA-Vitamin E-TPGS NPs (OA-PLGA-TPGS-NPs) was designed, evaluated, and compared for its value-added anti-leishmanial activity in comparison to SSG, PMC, and Amp-B against wild and resistant strains of *L. donovani*. The IC_50_ values of OA-PLGA-TPGS-NPs, 0.70 ± 0.10 μM and 6.80 ± 2.20 μM against *L. donovani* wild and SSG resistant strains, respectively, have clearly reflected enhanced leishmanial killing contrary to SSG and PMC. However, it also possesses slightly higher IC_50_ value than Amp-B against wild and resistant strains, which were 0.22 ± 0.07 μM and 0.26 ± 0.08 μM, respectively. Furthermore, these NPs possess valuable anti-leishmanial activity in *L. donovani* infected *BALB/c* mice with the reduction in spleen mass and splenic parasitic burden coupled with minimal nephrotoxicity (assessed using serum BUN and creatinine levels) [227]. Due to 19-times reduced IC_50_ than that of SSG, this nano-DDS could be of potential interest for detailed assessment in an in vivo setting. Nitric oxide possesses significant anti-leishmanial activity, however, its short half-life restricts its utilization. Incorporation of *S*-nitroso-mercaptosuccinic acid, a potent NO donor, inside chitosan NPs (NO-CNPs) has provided 99% release within 10 h. Moreover, 98% killing of *L. amazonensis* promastigotes was observed at 200 μM concentration, along with IC_50_ and IC_90_ were found to be 31.5 and 102.4 μM, respectively. MTT assay was also conducted to determine the safety of NO-CNPs and results indicated the unaltered mitochondrial activity of macrophages. Interestingly, immunofluorescence was also conducted and displayed with meaningful reduction of parasitophorous vacuoles, the residence of the parasite inside infected macrophages [228]. This study was highly valuable because it has utilized an un-conventional anti-leishmanial moiety, however, the researchers have not compared NO-CNPs anti-leishmanial activity with other drugs, which otherwise would have been more appropriate. The comparison of NO-CNPs with other nano-DDS, L-Amp-B, and conventional drugs (Amp-B, MA, etc.) could be conducted against Leishmania infected animal model to determine its utilization for further research.

## Leishmanial nano-vaccines—an innovative approach towards immunization

**Fig. 16:**
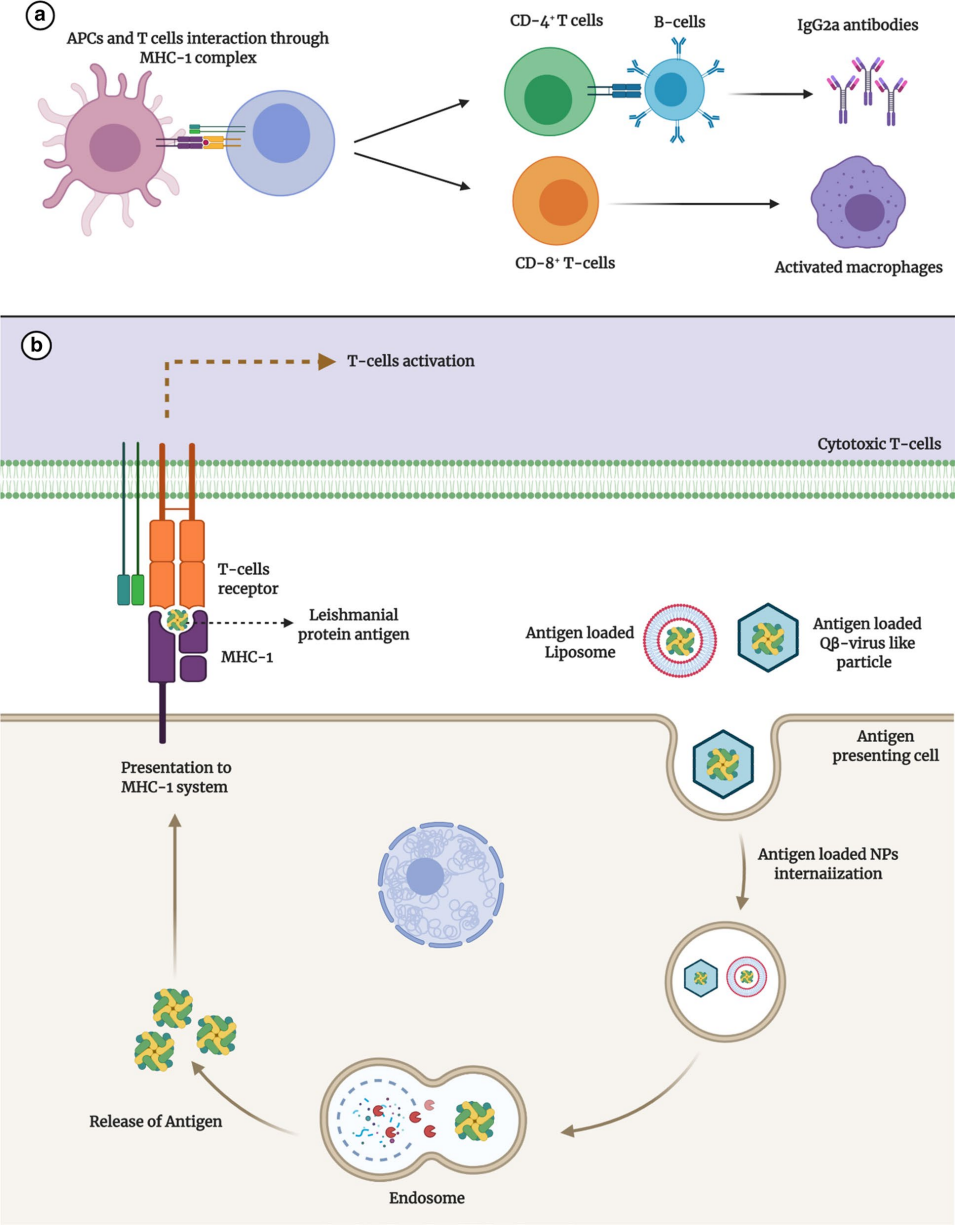
**a** Mechanism of adaptive immunity-APCs confronts the antigens to the T-cells through MHC-1 complex system followed by activation of macrophages and IgG2a antibody formation. **b** Nanosystem/Nano-adjuvants mediated leishmanial antigen protein delivery and activation of T-cells through MHC-1 and TCR complex

Leishmanial recombinant proteinaceous vaccines are, in fact, associated with poor activation of the MHC-I pathway and cell-mediated immunity. Virtues of nano-DDS and nanocarriers are not only limited to drug delivery. They can also be advantageous for vaccine delivery as well in order to induce an efficient immune response in the recipients. Utilization of peptide antigen-loaded nano-DDS is beneficial owing to shielded, safe, and adequate delivery of immunogenic molecule along with the capability of APCs internalization and innervation of T-helper cells-1 (Th-1) immune mechanism [229]. In other words, nanoparticle adjuvant—NPs capable of effective\antigen delivery to the APCs, is the very efficient approach towards potential immunization against this parasitic infection as illustrated in Fig. 16. Currently, none of the immunization is approved or marketed against Leishmaniasis. However, a promising immunization nominee (Heterogenous prime-boost) for Leishmaniasis, cysteine proteinase type-I, has been delivered to leishmanial infected mice using SLNs. This nano-DDS-based delivery of vaccine was found to be beneficial with the surge in immunomodulators along with parasitic burden reduction in C57BL/6 mice [230]. Another 2nd generation leishmanial vaccine has been evaluated for its in vivo response, followed by the administration in the form of P8-labelled soluble leishmanial antigen (SLA) loaded PLGA NPs. The PLGA nanocarriers were superficially modified using the sequence of 8 amino acids (P8) for active APCs targeting. The SLA was the active immunogenic moiety, hence considerable immune boost up was achieved in the *BALB/c* mice model [231]. At present, some vaccine candidates against Leishmaniasis are under pre-clinical and clinical trials [232]. Some of the limitations, with these under-trial vaccine nominees, can be resolved through the employment of these nano adjuvants or delivery systems.

### Liposomal adjuvant system for antigen delivery

LPS, as discussed erstwhile, are the promising nano-adjuvant for leishmanial antigen delivery to APCs. Antigen-loaded LPS are initially phagocytosed by the macrophages. These phagocytosed NPs are broken down inside lysosomes and antigen protein gets released in the cytoplasm. Which in turn, activates the endoplasmic reticulum or golgi apparatus major histocompatibility class-I and II (MHC-I and MHC-II) pathways. These events, however, activates cytotoxic T-cells response which numerous protein and non-replicating antigen-based vaccines lack [233]. Most of these nano-vaccines have proved their usefulness through the formation of IgG2a antibodies. Numerous factors should be considered while designing a liposomal adjuvant antigen delivery system because its immunogenicity has greatly been impacted by its nature and composition. Among them, the type of phospholipid used for LPS construction, the transition temperature of a phospholipid, liposomal surface charge, its size, and surface modification are the major ones. In general, antigen-loaded LPS having non-phosphatidylcholine phospholipids, phospholipids with higher transition temperature, larger size (above 100 nm), and positively charged surface can attain surged APCs uptake and internalization [234]. Most recently, LPS enclosing SLA and imiquimod has been assessed for its leishmanial immunization response in the *BALB/c* mice model. Mice experience reduces lesion size and footpad inflammation along with elevated blood levels of IgG2a and INF-γ, showing elevated T-helper cells-1 mediated immune response [235]. Other recently designed (in the past 6 years) liposomal based vaccine candidates are enlisted in Table 7.

**Table 7:** Recently designed nano-vaccines against Leishmaniasis

Nanosystem engineered	Core components for fabrication	Year	Loaded antigen	Animal model	Major results	Reference
Liposomes	DSPC + cholesterol	2019	Imiquimod + SLA	*L. major* infected *BALB/c* mice	Enhance CD-4^+^ and CD-8^+^ T cells response	[235]
	DPPC + cholesterol	2018	Recombinant LiHyR protein	*L. infantum* infected *BALB/c* mice	The increased concentration of IgG2a antibody was observed with significant parasitic burden reduction	[238]
	DOTAP* + cholesterol	2018	Recombinant glycoprotein (rgp63)	*L. major* infected *BALB/c* mice	Induce valuable immune response evidenced by a significant uptick in Th-1 cells mediated immune response and level of IgG2a antibody	[239]
	DDA + TDB + cholesterol	2017	SLA	*L. major* infected *BALB/c* mice	Inadequate protection against Leishmaniasis	[240]
	DOTAP + cholesterol	2016	Recombinant *Leishmania major* class I nuclease (rLmaCIN)	*L. major* infected *BALB/c* mice	Lowering in footpad lesion thickness among immunized subjects with significantly higher levels of IgG2a and INF-γ	[241]
	PC + SA + cholesterol	2015	Freeze thawed promastigotes	*L. donovani* infected *BALB/c* mice	Reduction in parasitic burden due to enhanced Th-1 cells response and associated cytokines	[242]
	DSPC + SA + cholesterol	2014	Recombinant cysteine protease mixture	*L. donovani* infected Syrian golden hamsters	Improved anti-leishmanial response with increased survival time and cytokines level however immune response was not adequately evaluated	[243]
	DSPC + cholesterol	2014	Thiolated antibody (IgG-SH) + SLA	*L. major* infected *BALB/c* mice	Improved Cytotoxic T-cells response	[244]
Virosomes	Qβ-virus like particles	2017	α-Gal trisaccharide	*L. amazonensis* and *L. infantum* infected C57BL/6 mice	The successful killing of parasites and eradication of infection evaluated through the RT-PCR technique	[245]
Polymeric NPs	PLGA	2021	SLA + TLR agonists	*L. major* promastigotes	Results have clearly shown improved macrophage activation accompanied by the significant reduction in pro-inflammatory cytokines	[236]
	PLGA	2020	SLA + Lipophosphoglycan	*L. infantum* infected *BALB/c* mice	Elevated NO, INF-γ, and IL-12 levels display 80% disease remission in infected *BALB/c*	[246]
	PLGA	2019	Recombinant proteins (rLpanUA.22 and rLpanUA.27)	*L. panamensis* infected *BALB/c* mice	Immune mediated parameters are not evaluated	[247]
	PLGA	2018	Recombinant proteins Chimeric peptides (CPA and CPB)	*L. major* infected *BALB/c* mice	Improved NO levels and cell-mediated immunity followed by immunization	[248]
	Chitosan	2018	Whole Leishmanial Lysate (WLL) + SLA	Leishmanial infected *BALB/c* mice	Poor immune protection was observed	[249]
	PLGA	2017	CPA	*L. infantum* infected transgenic mice	Enhance CD-4^+^ and CD-8^+^ T cells response Increased production of IL-12	[250]
	PLGA	2017	SLA with Toll like receptor (TLR-4) ligand	*L. infantum* infected *BALB/c* mice	Pronounced activation of cellular immunity due to enhanced Th-1 cells mediated immune response The levels of interleukins observed to be minimized (IL-6 and IL-10)	[231]
	PMMA	2016	pcDNA3	*L. major* infected *BALB/c* mice	Improve immune protection was observed clearly evaluated through uprise in IgG2a levels	[251]

### Polymeric NPs and virosomes for antigen delivery

Oral delivery of polymeric NPs is associated with M-cells interaction. M-cells traverse these NPs from the intestinal lumen to the APCs resident across the epithelium. Thus, polymeric NPs can be used as an antigenic carrier for mucosal immunization [90, 234]. Katebi et al. [236] loaded PLGA NPs with SLA and TLR receptor agonists—PAM3CSK4 and R848, to trigger macrophage-mediated phagocytosis against *L. major* parasites. Interestingly, results have shown a marked increment in macrophages phagocytotic potential accompanied by a significant reduction in pro-inflammatory cytokines. This immunogenic candidate should be tested against Leishmania infected animal model to determine in vivo parasitic and disease reduction potential [236]. Another innovative immunization technique is the use of viral-like particles or virosomes as a conveyance for antigen delivery. In contrast to conventional LPS, these are composed of pure glycoprotein viral coat embedded in the lipid bilayer membrane. This strategy not only improved the stability of the lipid bilayer but also promoted the significant improvement of immunogenicity. Influenza virus hemagglutinin and neuraminidase are usually intercalated inside phospholipid layers of virosomes [237]. Some of the recently designed virosomal and polymeric-based peptide nano-vaccines are mentioned and discussed in detail in Table 7. Numerous other nanosystems have also been utilized to evaluate their suitability for antigen/vaccine delivery against Leishmaniasis which includes SLNs, niosomes, micelles, and nanoemulsions.

## Nano-repellants and other nano-based strategies for vector control

For vector-borne infections, the transmission of the parasite can be contained by restricting the vector either by preventing its access to the healthy host or by killing it. Similarly, to control the spread of Leishmaniasis, different strategies may be employed including the use of repellents, insecticides treated nets, and anti-parasitic infestation drug utilization by host organism [252]. The use of chemically derived repellants is a practical approach for Leishmaniasis prevention owing to the unavailability of effective vaccination, but they have the potential to cause toxicity with protracted use. Diethyl-toluamide (DEET), diethyl phthalate, and diethyl-phenyl acetamide (DEPA) possess considerable repellant properties for phlebotomine sandflies [253]. Here, nanotechnology was employed to provide longevity of repellant action, impeding its evaporation rate and reduction in its toxicity. Among currently marketed repellants, DEET is the most effective one and considered as a standard for comparison purposes as well. Due to excessive use, unfortunately, its efficacy is declining considerably [78]. A group of scientists has loaded DEET inside polymerically-poly-methacrylate (PMMA), constructed nanospheres and interestingly, this nano-repellant appear to attain the sustained release properties with prolonging the duration of action up to 9 h, significantly enhanced as compared to plain DEET. Thus, its excessive use can be tackled by employing nano-DEET [254]. Permethrin (PMT) has also been loaded inside lipid-based nanosized carriers and applied on clothes as a repellant. This technique was found to be advantageous over free PMT because nanoparticles stuck to the polyester fibers even after washing. Thus, the utilization of PMT-NPs, exhibiting improved repellant effect, can prevent insect bite and parasitic transmission from diseased to healthy host [255]. In another study, DEPA entrapped PEG NPs were prepared by Balaji et al. [256]. This nano-repellant has been found to exhibit 5-times depression in LC_50_ value as compared to conventional DEPA repellants. Moreover, their impregnation in cotton clothes also appeared with long-lasting repellant nature. To sum up, myriad attempts have been made to fabricate nano-repellants. However, with context to Leishmaniasis, none of them have been evaluated against phlebotomine species for their improved repellant actions. This is a critical point that needs to be addressed by scientists and nano-engineers on an immediate basis. Indoor pesticide sprays of dichloro-diphenyl-trichloroethane (DDT) were, initially, found to be adequately effective in controlling the Leishmaniasis vector, the phlebotomine sand-fly. A study carried out in 2016, to assess the resistance incidence among *P. argentipes* and *P. papatasi*, has found that DDT has become less effective in several areas of India, Sri lanka, and Bangladesh [257]. To the best of our knowledge, no study is available yet that has designed the DDT nanoparticles—a possible way to improve DDT efficacy against sand-fly species. Despite their usefulness as a delivery system, metallic NPs (Au-NPs, Ag-NPs, and ZnO-NPs) have also gained interest in their application for vector control. Particularly, the naturally-obtained extract-loaded metallic NPs are proved to exhibit ovicidal and larvicidal properties when obtained through the green synthesis process. These NPs are beneficial in terms of biocompatibility and good safety profile [78, 258]. Still, there is a lack of evidence about their usefulness against *Phlebotomus* flies and tested only for mosquito (*A. egypti*) control [259]. These studies should be necessarily conducted in regions where Leishmaniasis is endemic to obtain nanotechnology-based controls against Leishmaniasis vector.

## Loopholes in designed nano-DDS

### Nanotoxicity—a critical safety concern, and its assessment

Nanomedicine is achieving high milestones and further advancement of this horizon is in progress. Thus, because of their valuable gains, the number of FDA approvals is constantly escalating [260, 261]. Due to the rise in their post-market acceptability, there is a crucial requirement to develop accurate methods and tests for the safety or toxicity assessment of nanoparticles. According to our literature survey, the field of nanotoxicity assessment is obscure, and very limited information is available. FDA as a global health authority should require devising the standard testing methods and procedures for nanomaterials toxicity assessment because the conventional safety analysis techniques are not suitable for nano-formulations owing to their distinctive nature, size, and surface properties [262]. It has been evidenced by results variation in a study carried out by Clift et al. [263] showing that nanofibers induced genotoxicity is perceptible through mammalian assay but not with conventional Ames assay. At present, the toxicity of engineered nanomaterials is assessed by several in vitro and in vivo techniques, both of which are associated with certain drawbacks in context to NPs toxicity measurement. As a response, there is a requirement to develop some valuable and accurate QSAR-based in-silico techniques, on an immediate basis [264].

#### In vitro nanotoxicity assessment

Inflammation, cytotoxicity, immunotoxicity, genotoxicity, carcinogenicity, and hematotoxicity are the frequently evaluated parameters through in vitro analysis, represented in Table 8 [265]. In vitro assays are mainly employed as they are less time-consuming and low budgeted. Cytotoxicity assessment of NPs is mainly carried out either by determining the presence of apoptotic/necrotic cells using Annexin-V assay and the proportion of apoptotic/necrotic cells by flow cytometry and cell viability using the most commonly used MTT assay. Moreover, the cell membrane integrity and cell morphology can be examined via lactate dehydrogenase (LDH) assay and phase-contrast microscopic evaluation, respectively [262, 265]. Unfortunately, nano-DDS have been found to exhibit the interference with aforementioned toxicity assays along with excessive chances of false results [266]. Nanoparticles, specifically, lipid-based nano-DDS when administered via intravenous route face-off the body intrinsic defense system i.e. macrophages, and other components of RES. Certain opsonizing complement proteins mark the NPs which can easily be trapped by phagocytic cells. All these events eventually cause either the destruction of engineered NPs followed by reduced NPs build-up in targeted tissue or the activation of the body’s immune response. Type-1 hypersensitivity response is a frequently encountered immune response associated with NPs [266–268]. This would lead to a life-threatening anaphylactic shock. With all the discussion, it can be concluded that the immunological assessment of designed nano-DDS is a critical requirement. Inflammation can easily be assessed by quantifying the inflammatory markers such as TNF-α, IL-6, IL-8, complement proteins, and glycoproteins like granulocyte macrophage-colony stimulating factor (GM-CSF) using the method referred to as Enzyme-linked immunosorbent assay (ELISA) [268, 269]. These pro-inflammatory indicators are also analyzed by their level assessment in human peripheral blood monocytes and lymphocytes [270]. Some nanomaterials are potentially damaging to genetic material as well. This type of toxicity is also concerning and should be routinely examined. Previously Ames assay was utilized, but due to the high incidence of false results mammalian cell, the micronuclear assay has replaced its employment [265, 271]. Furthermore, for NPs associated carcinogenicity detection, the colony transformation assay (CTA) is employing currently [272]. Nanomaterials used for infections and cancer also cause the production of ROS in normal cells. This could possibly lead to cell destruction and toxicity. Apart from erstwhile mention cytotoxic analysis, some other techniques are employed for evaluation of ROS genesis inclusively ferric reduction ability of serum (FRAS) assay, NO evaluation and the use of fluorescent or non-fluorescent markers i.e. dichloroflorescein [262, 273].

**Table 8:** Parameters and frequently employed assays for nanotoxicity assessment

Parameter to be assessed	Type of analysis	Suitable experimental model/assay	Limitations/comments	Reference
Cytotoxicity	Apoptosis and necrosis	Annexin-V coupled with propidium iodide	CNTs are inappropriate for MTT evaluation because of their interference and possible false outcomes Possibly, the concentration or dose employed for testing is influential towards the test results Due to limitations of MTT assay, Flow cytometry and Annexin-V coupled with propidium iodide are the appropriate options	[262, 266]
	Cell death determination	Flow cytometry		
	Cell proliferation	Colony-forming efficiency assay		
	Cell viability and altered metabolic activity	MTT/WST-1 assay + colorimetric assay using Alamar blue dye		
	Cell membrane integrity	LDH assay		
	Cell morphology assessment	Phase-contrast microscopy		
	Epithelial cell damage	Impedance based measurements		
Inflammatory response	Inflammatory markers (cytokines, chemokines and other proteinaceous markers)	ELISA + cytokines determination in human peripheral blood mononuclear cells	Higher doses of NPs could cause cell lysis, which ultimately reduces the actual levels of inflammatory markers. Thus, the chances of false (underestimated) results are high Metallic, silica NPs and CNTs show this type of interference	[265, 266, 295]
Genetic toxicity	Genetic material damage	Ames assay + mammalian cell micro-nuclear assay	Nanofibers induced genotoxicity is precipitable through mammalian assay but not with conventional Ames assay due to interference with results	[265, 271]
Oxidative stress	Evaluation of ROS	FRAS assay + nitric oxide evaluation + fluorescent or non-fluorescent markers i.e. dichloroflorescein	Interference of MWCNTs, TiO_2_ NPs, Iron oxide NPs and graphene-based NPs with probe (fluorescent or non-fluorescent marking) method triggers the false results	[262]
Carcinogenicity	Carcinogenic potential	Colony transformation assay (CTA) + *BALB/c* 3T3 cell transformation assay + syrian hamster embryo assay	This technique can determine both genotoxic along non-genotoxic carcinogenicity However, the “gold standard” method for NPs carcinogenicity evaluation is assessment in Laboratory animals (In vivo experimental model)	[272]

#### In vivo toxicity assessment

The biological organisms consist of a highly complex system and administered NPs could interact with various biological molecules. The in vivo animal models are the suitable option for NPs toxicity quantification in the biological system. For immunotoxicity, I.V. injection of fabricated nanosystem to the porcine model is most efficient. Thus, NPs with the potential to induce type-1 allergic response, predominantly LPS, are evaluated by this method [265]. In vivo rat's skin irritation test works on the same principle but utilized particularly for topically and transdermally administered nano-formulations [274, 275]. Carcinogenicity of NPs evaluations in laboratory animal models is, in fact, a standard test. However, due to high cost, excessive time consumption, and using plenty of animals, the previously mentioned in vitro experiments are currently being utilized [272]. Imaging techniques like optical imaging, microscopy MRI [276], CT [277], and PET scanning, are also the suitable options for real-time monitoring of NPs fate and toxicity in vitro as well as in vivo [278, 279].

#### Toxicity coupled with various nano-DDS

With the expansion of nanomedicine, the knowledge about its harmful effects is also growing at a steady pace. LPS, the most widely used lipid nanomaterial is associated with a serious toxic effect—an anaphylactic shock. This incidence was considerably high with conventional LPS; however, the PEGylated LPS have reduced the chances [280, 281]. The damaging impact of SLNs over the biological system usually depends upon the ingredient i.e. the lipid and the surfactant, employed for their synthesis [282]. Furthermore, other nano-DDS utilizing surfactants are also considered to be toxic in nature [283]. Polymeric NPs, because of using biologically compatible and natural polymers, are considered to be safe, however, toxicity needs to be evaluated properly [284]. Metallic NPs also express some unwanted effects. They are often associated with cytotoxicity, particularly hepatotoxicity [285–288] and neurotoxicity [289, 290], due to the production of ROS, free radicals, mitochondrial damage, and cell death [291, 292]. Generally, the size of nano-DDS is, merely, a critical parameter that assesses the toxicity profile—the smaller the size, the more it will be toxic. Thus, the designed NPs must have a size in the adequate range. Similarly, Carbon nanotubes induced cytotoxicity has also gained attention for further investigations and explorations [293, 294]. The parallel emergence of nano-toxicology with nanotechnology is urging the pharmaceutical scientists and researchers to opt naturally and plant derived nanomaterials, aiming towards minimization of “synthetics” use.

### Economic burden—lack of cost-effectiveness

Unfortunately, nano-formulations (nano-therapeutics, nano-diagnostics, and nano-vaccines) usually hold high research, testing, and manufacturing cost. By 2018, Europeans and American institutes have invested about €430 million and $445 million, respectively for nanomedicine research. These economic constraints have made, the price posing of nano-formulation, a quite challenging task for pharmaceutical companies. Nano-formulations are, indeed, considerably high-priced than traditional dosage forms. There should be adequate cost-effectiveness analysis of nano-formulation, for the purpose to minimize this market hampering factor. Only by this way high selling prices of nano-products could be contained [296].

### Marketing and commercialization challenges

Nanopharmaceuticals are difficult to scale up due to the complexity of ingredients and the manufacturing process. Approval and commercialization of nanopharmaceuticals are hindered by many parameters discussed in this paragraph. Despite the aptness of LPS, some shortcomings are also associated with these lipid-based nanocarriers. Leakage of the entrapped moiety, oxidation, hydrolysis, and inadequate stability are the drawbacks of liposomal nano-DDS [297]. Though SLNs have confronted the erstwhile stated stability enigma, but their employment has also been linked with several setbacks. Inadequate loading efficiency and expulsive release of the entrapped drug, during storage, owing to lipid phase transition are among them. NLCs were thus designed as a solution of SLNs associated negatives [282, 298, 299]. As far as polymeric NPs are concerned, they are inconvenient to scale-up [300]. The hurdles, related to these most commonly employed nano-DDS [260], can be encountered by further investigation and research as these are, perhaps, the hurdles for nanomedicine’s market compliance. Generally, the dissimilar in vivo and in vitro behavior of nano-formulations is also a major contributory factor towards their compact market. Proper animal and biomedical testing facilities are required which are not easily achievable and non-cost effective. Some regulatory and ethical problems are also hindering the nanosystem’s trackway towards commercialization. FDA and European medicines agency (EMA) have imposed un-necessary (sometimes) and excessive regulatory restrictions, ultimately, restraining the approval process. Globally, pharmaceutical industries are not much interested in this novel medical treatment approach. Research institutes and academia are majorly involved in nanomaterial research. However, due to poor academia-industry collaboration, limited testing facilities, and insufficient funding, very little by the way of nanosystem reach towards successful commercial utilization [301].

## Future perspective

With advancements in nanotechnology and nano-DDS, the associated loopholes and nanotoxicity are demanding more scientific attention and need to be addressed. Mannosylated thiolated nano-formulations, through the evaluation of available literature, are found to be the most promising nano-DDS for Leishmaniasis. However, researchers and scientists should identify some newer metabolic and enzymatic targets—metacaspases, folate biosynthesis, protein kinases, etc., for parasitic curtailment [77]. Leish-111F is having efficacy issues in clinical trials and probably, by the utilization of nano-adjuvants (discussed in “Leishmanial nano-vaccines—an innovative approach towards immunization” section), its efficacy would be enhanced. Drug loaded phyto-nano-DDS could also be an appropriate option to tackle Leishmaniasis. After critically evaluating the research work related to anti-leishmanial nano-DDS, on preclinical grounds, several limitations have been identified. In numerous studies, in vivo anti-leishmanial assay was not performed. In addition, various nano-DDS research reports were not properly compared with standard anti-leishmanial therapies. It is worth mentioning that standard protocols for preclinical evaluation of nano-DDS should be designed in terms of species and strains of the animal to be employed, parasite species and strains, duration of the study, and reference standard by which the anti-leishmanial activity of nano-DDS can be determined comparatively. By employing these standard protocols uniformity, precision, and robustness of results can be achieved.

Multiple anti-leishmanial nano-DDS with promising results on pre-clinical grounds, must be step up for human clinical studies. According to NIH clinical trial repository, only L-Amp-B has been enrolled against VL, with a trial identifier ID of NCT02025491 and NCT00628719 [302, 303]. However, to improve treatment outcomes of CL, MA-LPS, PMC-LPS, and L-Amp-B have been enrolled in clinical trial (identifier number NCT01050777 and NCT02656797) [304, 305]. Though, there is a fall short of human studies for the evaluation of safety and efficacy of the anti-leishmanial nano-DDS. Probably, with significant academia and pharma industry collaboration, nanotechnology-based drug delivery of anti-leishmanials will eventually be studied in more detail, with a few of the promising candidates will get enrolled in clinical trials for the determination of their efficacy and safety in the human population. Antigenic protein delivery using nano-adjuvants will hopefully emerge some promising immunogenic candidates for immune-based protection against Leishmaniasis. Another important aspect, the cost-effective scaling up of nano-DDS, should require significant attention and improvement. Devising a global policy to provide cost-effective nano-DDS, is the only way to excel towards nanotechnology-based eradication strategies against Leishmaniasis. Nevertheless, approval of cost-effective anti-leishmanial nano-formulations could be speculated to make treatment more effective and shorter in duration. Alongside, total to partial extermination of this disease can be sought, along with associated co-morbidities.

## Concluding remarks

Apart from anti-leishmanials, 28 nano-DDS have attained FDA/EMA approval to date [261, 306]. Extensive nanotechnology based research has carried out to rationalize Leishmaniasis management in all dimensions. Drug loaded nano-DDS have been found to curtail various challenges evidenced by in vitro and preclinical testing. In the last decade, far too many anti-leishmanial nano-DDS have been formulated and tested on animals for the claimed resolution of limiting factors. The most problematic issue encountered during Leishmaniasis treatment, inadequate intracellular accumulation of drug, can effectively improve by employing phosphatidylserine labeled and mannosylated nanoparticles which in turn have a positive impact on treatment prognosis in animal models. Their superiority over plain nanoparticles could clearly be evidenced by multiple studies. Leishmanial resistance, however, can be countered through thiolated nano-DDS, and co-loaded nano-DDS. Remolding conventional routes of administration could also be gained by using a nano-drug delivery-based approach. Similarly, NPs encapsulating the drug, on one hand, improve pharmacokinetics constraints of the drug and on the other hand minimize its exposure to un-infected body cells, thereby reducing drug toxicity. Emerging advancements in LPS and TFS delivery could be an efficient tool in targeting the cutaneous form of Leishmaniasis. With the identification of L-Amp-B resistant case, there is a need of the hour to work more aggressively over co-loaded and targeted nano-formulations for the purpose to prevent further resistance emergence, making the treatment more challenging. Particularly, due to lack of academia and pharmaceutical industry collaboration, very little efforts are so far done to launch the nano-DDS in the market. Phyto-nano-DDS are also gaining attention to curb Leishmaniasis because of their extra-safe nature. Vaccination for Leishmaniasis is under the process of development, and obstacles in the way of effective immunization are being tried to cope with using nanovaccines—using NPs for effective antigen delivery to APCs. In endemic regions, there should be required evaluating the effectiveness of nano-repellants and nano-insecticides in restricting the disease spread via vector control strategy. The discovery of new and more effective agents is deemed to be necessary because of certain reasons but, as mentioned erstwhile, drug discovery is a protracted bid to improve Leishmaniasis treatment and eradication. In addition, chemical and functional group manipulation of existing drugs also appear to possess the same restrictive parameters. Therefore, nanoscale-advancement in drug delivery is the most appropriate approach to attain desired goals. In a nutshell, nano-DDS based formulations, are the groundbreaking approach towards the anti-leishmanial therapeutic advancement and success.

## Data Availability

Not applicable.

## Abbreviations

ABC transporter: ATP-binding cassette transporter
AgNPs: Silver nanoparticles
Amp-B: Amphotericin-B
APC: Antigen presenting cells
ATP: Adenosine triphosphate
AUC: Area under curve
AuNPs: Gold nanoparticles
AuNPs: Gold NPs
BCS: Biopharmaceutics classification system
BSA: Bovine serum albumin
CC_50_: Concentration to kill 50% of the cells (cytotoxic concentration)
CL: Cutaneous Leishmaniasis
CLSM: Confocal laser scanning microscopy
C_max_: Maximum concentration
CNTS: Carbon nanotubes
CTA: Colony transformation assay
CUR: Curcumin
DDA: Dimethyl-dioctadecyl ammonium
DDT: Dichloro-diphenyl-trichloroethane
DEET: Diethyl-toluamide
DEPA: Diethyl-phenyl acetamide
DPPC: Dipalmitoyl phosphatidylcholine
DSPC: 1,2-Distearoyl-sn-glycero-3-phosphocholine
ED_50_: Median effective dose
ELISA: Enzyme-linked immunosorbent assay
EMA: European medicines agency
f-CNTs: Functionalized carbon nanotubes
FDA: Food and Drug Administration
FRAS: Ferric reduction ability of serum
FTIC: Fluorescein isothiocyanate
GIT: Gastrointestinal tract
GM-CSF: Granulocyte macrophage-colony stimulating factor
IC_50_: Concentration to inhibit micro-organism growth by 50%
IL-12: Interleukin-12
IL-6: Interleukin-6
IL-8: Interleukin-8
IMQ: Imiquimod
L-Amp-B: Liposomal amphotericin B
LC_50_: Concentration to kill 50% of organisms
LDH: Lactate dehydrogenase
LDL: Low-density lipoproteins
LPS: Liposomes
MA: Meglumine Antimoniate
M-cells: Microfold cells of the intestine
MCL: Mucocutaneous Leishmaniasis
MFS: Miltefosine
MHC-I: Major histocompatibility complex-I
MHC-II: Major histocompatibility complex-II
MIC-: Minimum inhibitory concentration
MK-TFS: Miltefosine-ketoconazole co-loaded transfersomes
MSN: Mesoporous silica NPs
MTC: Mannosylated thiolated chitosan
MWCNTs: Multi-walled carbon NPs
Nano-DDS: Nano drug delivery system
NLCs: Nanostructured lipid carriers
NPs: Nanoparticles
PC: Phosphatidylcholine
PCL: Poly-caprolactone
PEG: Polyethylene glycol
PET: Positron emission tomography
P-gp: P-glycoprotein
PLGA: Poly-lacto-glycolic acid
PMC: Paromomycin
PMMA: Polymethyl methacrylate
PMQ: Primaquine
PMT: Permethrin
PRP-1: Pentamidine resistant protein-1
PTM: Pentamidine
QSAR: Quantitative structure–activity relationship
RES: Reticuloendothelial system
RMQ: Resiquimod
ROS: Reactive oxygen moieties
SA: Stearylamine
SC: Stratum corneum
SLA: Soluble leishmanial antigen
SR: Scavenger receptor
SSG: Sodium Stibogluconate
TDB: Trehalose-6,6-dibehenate
TFS: Transferosomes
Th-1: T-helper cells 1
TiO_2_: Titanium oxide NPs
TNF-α: Tissue necrosis factor-α
TR-system: Trypanothione reductase system
USD: United States dollar
VL: Visceral Leishmaniasis
WHO: World Health Organization
ZnO-NPs: Zinc oxide NPs
